# Rapid *in situ* encapsulation of [NiFe]-hydrogenase into covalent organic frameworks for robust hydrogen oxidation and evolution

**DOI:** 10.1039/d6sc01349j

**Published:** 2026-05-25

**Authors:** Islam E. Khalil, Stefan Frielingsdorf, Prasenjit Das, Christian Lorent, Christian Teutloff, Augustine A. Owusu, Armel F. Tadjoung Waffo, Ingo Zebger, Oliver Lenz, Arne Thomas

**Affiliations:** a Department of Chemistry, Functional Materials, Technische Universität Berlin 10623 Berlin Germany; b Department of Chemistry, Biophysical Chemistry, Technische Universität Berlin Straße des 17. Juni 135 10623 Berlin, Germany; c Department of Physics, Freie Universität Berlin Arnimallee 14 14195 Berlin Germany; d Department of Chemistry, Technische Universität München Lichtenbergstr. 4 85748 Garching Germany arne.thomas@tum.de

## Abstract

[NiFe]-hydrogenases are highly efficient metalloenzymes capable of reversibly converting hydrogen gas into protons and electrons, positioning them as valuable catalysts for sustainable hydrogen production and bioenergy systems. However, their practical deployment is limited by poor stability and the difficulty of integrating them into solid supports without compromising activity. Covalent organic frameworks (COFs) offer a promising platform for enzyme immobilization due to their high surface area, tunable pore environments, and robust chemical stability. These features can improve enzyme loading, protect structural integrity, and enhance overall catalytic performance. Here, we report a rapid *in situ* encapsulation strategy that embeds a membrane-bound [NiFe]-hydrogenase (MBH) directly into the network of a 1,3,5-triformylphloroglucinol (Tp) and *p*-phenylenediamine (Pa) β-ketoenamine COF (denoted MBH@TpPa) within 10 min. This approach bypasses multistep post-synthetic procedures typically required for enzyme immobilization and preserves the intrinsic activity of the enzyme. The resulting MBH@TpPa biohybrid exhibits outstanding photocatalytic hydrogen evolution activity, achieving 2.59 mmol g^−1^ h^−1^, a 43-fold enhancement over the pristine COF and more than three times the performance of Pt-loaded TpPa. In addition, MBH@TpPa catalyzes sustainable hydrogen oxidation, a more demanding transformation, demonstrating its bidirectional reactivity. These results establish MBH@TpPa as a versatile and robust biohybrid catalyst with significant potential for solar-driven and renewable energy applications.

## Abstract 2

The rapid *in situ* encapsulation of a [NiFe]-hydrogenase into covalent organic frameworks (COFs) enables noble metal-free photocatalytic hydrogen evolution and hydrogen oxidation reactions.

## Graphical abstract

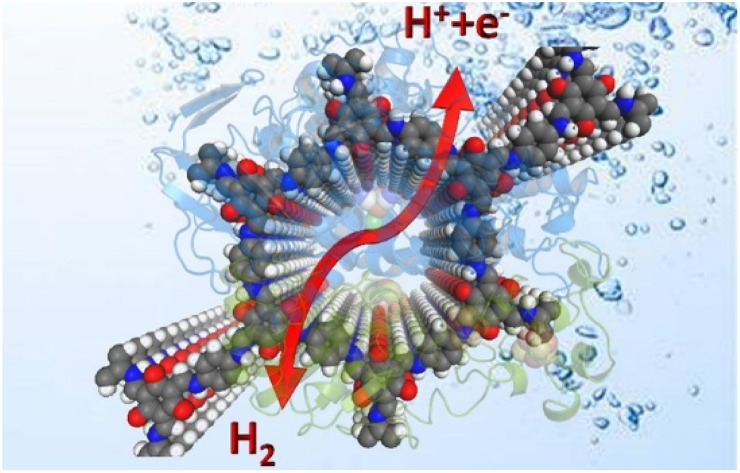

## Introduction

Hydrogenases are metalloenzymes that catalyze the reversible conversion of molecular hydrogen (H_2_ ⇌ 2H^+^ + 2e^−^) at a dinuclear metal center, where heterolytic H_2_ cleavage is facilitated and the liberated electrons are transferred *via* a conserved relay of Fe–S clusters.^1^ Although these enzymes are theoretically bidirectional, they typically exhibit a pronounced catalytic bias. Though many [FeFe]-hydrogenases are highly efficient in H_2_ evolution, most of these enzymes are irreversibly deactivated by trace amounts of oxygen.^2^ In contrast, oxygen-tolerant [NiFe]-hydrogenases, such as the membrane-bound hydrogenase (MBH) from *Cupriavidus necator*, perform catalysis in the presence of O_2_, but predominantly catalyze H_2_ oxidation and show only limited proton reduction activity.^3^ This intrinsic bias poses a challenge for their integration into bidirectional energy conversion systems, including fuel cells and electrolyzers. While MBH's oxygen tolerance is advantageous for industrial applications, its low proton reduction activity under ambient conditions remains a key limitation.^4^ MBH-type hydrogenases can easily be contacted by conductive materials because the Fe–S cluster most distal to the [NiFe] site is located about 8 Å from the protein surface,^5^ and immobilizing hydrogenases on solid-state scaffolds has therefore emerged as an effective strategy to improve their stability, prolong operational lifespan, and enhance resistance to deactivation.^6–8^ A notable example is the [NiFeSe]-hydrogenase from *Desulfomicrobium baculatum* immobilized on Ru dye-sensitized TiO_2_ nanoparticles, which achieves a turnover frequency of ∼50 s^−1^ under visible light while maintaining activity even after air exposure. Despite its robustness, this system relies on TiO_2_, expensive Ru-based photosensitizers, and lacks covalent enzyme anchoring.^6^ Nevertheless, such enzyme-material hybrids exemplify the promise of integrating biocatalysts into engineered scaffolds to combine the selectivity of biological systems with the tunability of synthetic materials. Among the various supports investigated, metal–organic frameworks (MOFs) have garnered substantial interest due to their structural tunability, chemical diversity, and high surface areas.^9–11^ However, concerns regarding the potential release of toxic metal ions and insufficient stability under harsh chemical or aqueous environments have limited their broader applicability.^12,13^ Consequently, the development of metal-free, chemically resilient, and structurally ordered materials is of growing interest. Recent efforts have explored hydrogen-bonded organic frameworks (HOFs) as alternative crystalline matrices for protein encapsulation.^14–16^ Nonetheless, the inherently weak intermolecular interactions within HOFs can compromise their durability and recyclability.^17,18^

In this context, covalent organic frameworks (COFs), crystalline porous polymers constructed from organic building blocks linked by strong covalent bonds, have attracted increasing attention as advanced platforms for enzyme immobilization. Their inherent structural order, tunable pore geometries, and modular functionality offer unique advantages for hosting biomacromolecules in catalytically active environments.^19,20^ This stands in contrast to conventional amorphous polymers, which typically lack defined porosity. These are usually prepared with (noble) metal catalysts, organic solvents and/or elevated temperatures, reaction conditions which are not compatible with sensitive metalloenzymes such as [NiFe]-hydrogenases. For enzyme entrapment to be effective, the support material must meet several critical requirements: it must exhibit chemical and structural stability under operating conditions, possess pores large enough to facilitate diffusion of substrates, products, and (co)enzymes, and be synthesized under mild conditions to preserve enzymatic activity.^21^ While COFs fulfill the first two criteria exceptionally well, their synthesis typically requires elevated temperatures or organic solvents, conditions that are incompatible with most proteins, thus precluding straightforward one-pot encapsulation.^22,23^ Consequently, post-synthetic strategies such as physical adsorption are commonly employed; however, these approaches often suffer from low enzyme loading and high leaching, stemming from weak host–guest interactions or mismatched pore dimensions.^24^

To address these challenges, a range of advanced strategies has been explored, including the incorporation of reactive functional groups for covalent anchoring, the use of enzyme-loaded MOFs as sacrificial templates, and the rational design of pore architectures tailored to enzyme size.^19,25–27^ For instance, our group recently demonstrated that a [NiFe]-hydrogenase (MBH) can be encapsulated within a β-ketoenamine COF functionalized with sulfonic or carboxylic acid groups. Because the size of the enzyme far exceeds the intrinsic pore size of the COF, additional macropores had to be introduced into the framework using a polystyrene template, allowing the enzyme to be accommodated within these larger cavities. This architecture enabled efficient enzyme immobilization and facilitated direct bioelectrocatalytic H_2_ oxidation without mediators, while also dramatically enhancing the enzyme's operational stability over a period of 14 days.^28^ Although this represents a significant step forward for COF/MBH assemblies, the strategy still relies on a relatively complex, multistep fabrication procedure, which limits scalability and practical applicability. Moreover, enzyme leaching from the large pores over extended operation times cannot be entirely excluded. Recent advances in *in situ* COF encapsulation strategies demonstrate their potential for enzyme stabilization and activity enhancement.^29–32^ For example, Liang and colleagues prepared COF-LZU1 in water at room temperature and achieved high retention of the encapsulated horseradish peroxidase (HRP) activity. Their study demonstrated COFs as versatile hosts for encapsulating multiple enzymes and protecting them under harsh conditions, though it centered on hydrolytic biocatalysts with limited industrial scope.^33^ Recently, the Chen group reported a 10 minutes synthesis of mesoporous COFs to covalently anchor lipase for chiral resolutions. A solar-driven photothermal effect enhanced lipase activity 2.5-fold.^34^ Despite these advances, both approaches remain confined to mild hydrolytic processes, leaving larger, sensitive metalloenzymes such as a hydrogenase unexplored in COF matrices. This gap underscores the need for tailored encapsulation strategies that can accommodate the unique structural and catalytic demands of advanced metalloenzymes under simple, scalable conditions.

In this work, we developed a rapid, aqueous-phase one-pot synthesis of a β-ketoenamine COF (TpPa) tailored for encapsulation of the membrane-bound hydrogenase (MBH) from *Cupriavidus necator*. Obviously, an enzyme such as MBH, with a size of approximately 8 nm, cannot penetrate or reside within the micropores of a COF with diameters not exceeding 1.75 nm. Consequently, a strategy was adopted in which the framework material is formed *in situ* in the presence of the enzyme, growing around it in a cage-like manner, encapsulating and thereby stabilizing the biomolecule. During this process, the enzyme effectively creates its own cavity within the growing framework, while the intrinsic micropores of the surrounding network ensure the accessibility of reactants to the enzyme and the efficient removal of products. To the best of our knowledge, such *in situ* encapsulation of a large and highly sensitive metalloenzyme has not been attempted to date and naturally poses significant challenges for the conditions required for framework synthesis.

**Fig. 1:**
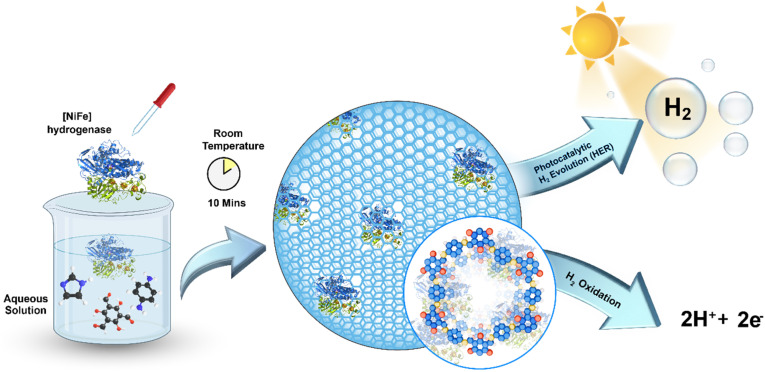
Schematic representation of the one-pot, aqueous-phase synthesis where the TpPa COF grows around the membrane-bound hydrogenase (MBH), leading to the formation of the MBH@TpPa biohybrid.

To achieve this, a dissolution-precipitation approach was employed, enabling COF crystallization in water at room temperature within 10 min. Imidazole was used to enhance the solubility of the organic monomers, while ethanol, being miscible with water and capable of hydrogen bonding, served as a precipitant, thereby promoting rapid crystallization with high structural quality. The resulting MBH@TpPa hybrid retained the enzyme's oxygen tolerance while significantly enhancing its proton reduction activity. Under photocatalytic conditions, MBH@TpPa achieved a hydrogen evolution rate (HER) of 2.59 mmol g^−1^ h^−1^, a 43-fold enhancement over the pristine COF (0.06 mmol g^−1^ h^−1^), demonstrating the synergistic effect of enzymatic active sites and COF-mediated charge transfer. Intriguingly, the performance is still more than three times higher than that of the TpPa COF loaded with platinum (Pt) (0.81 mmol g^−1^ h^−1^). This performance highlights the dual advantage of avoiding costly Pt while leveraging MBH's inherent catalytic bias, which is amplified by the COF's ordered pores and light-harvesting properties (Fig. 1). This work shows that rapid, aqueous-phase encapsulation of the complex [NiFe]-hydrogenase into a β-ketoenamine COF creates a biohybrid photocatalyst that outperforms even platinum-based benchmarks, showcasing a scalable route to high-efficiency, noble-metal-free systems for photocatalytic HER.

## Result and discussion

### Synthesis and characterization of encapsulated hydrogenase in COF

**Fig. 2:**
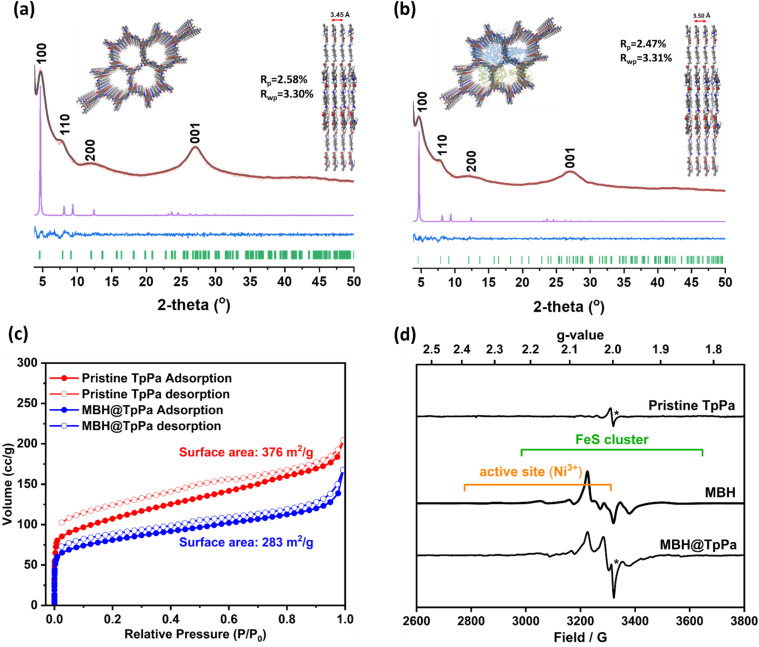
Pawley refined (red dotted line), experimental (black line) PXRD pattern with minimum difference (blue line) for hexagonal AA stacking (purple) of (a) pristine TpPa and (b) MBH@TpPa. (c) N_2_ sorption isotherms of pristine TpPa and MBH@TpPa COFs at 77 K. (d) EPR spectra of pristine TpPa COF (top), MBH solution (middle) and MBH@TpPa COF (bottom) recorded at 20 K. The spectrum of MBH solution is scaled to fit the intensity of the signal from MBH@TpPa COF. The asterisk highlights an organic radical signal from the TpPa COF.

In accordance with previous reports,^35^ the MBH@TpPa COF was synthesized *via* a Schiff base condensation reaction, with modifications to improve monomer solubility and facilitate subsequent functionalization. Specifically, 1,3,5-triformylphloroglucinol (Tp) and *p*-phenylenediamine (Pa) were employed as the aldehyde and amine monomers, respectively, in the presence of imidazole to enhance solubility in aqueous media. The reaction was carried out at ambient temperature. Prior to the introduction of MBH for immobilization, the solution pH was adjusted to 5.5 by the addition of acetic acid as MBH prefers slightly acidic conditions. The resultant COF was precipitated using ethanol, as detailed in the SI.^35^ To test the effect of EtOH on MBH, the enzyme was incubated with EtOH in buffer under the same conditions and an activity reduction of only 8.2% was observed. The crystallinity of pristine TpPa and MBH@TpPa was investigated *via* powder X-ray diffraction (PXRD). Both materials exhibited a prominent diffraction peak at 2*θ* = 4.78°, corresponding to the (100) reflection of an ordered two-dimensional hexagonal lattice. Secondary peaks observed at 2*θ* ≈ 8.17–12.12° were assigned to the (110) and (200) planes, while a broad peak centered at 2*θ* = 27.1° was attributed to the (001) reflection, indicative of π–π stacking interactions between adjacent layers. Structural simulations demonstrated good agreement with an eclipsed AA-stacking configuration. Pawley refinement of the experimental patterns yielded low residuals (pristine TpPa: *R*_wp_ = 3.30%, *R*_p_ = 2.58%; MBH@TpPa: *R*_wp_ = 3.31%, *R*_p_ = 2.47%), confirming the reliability of the proposed structural model. The refined unit cell parameters were consistent with a hexagonal lattice (space group *P*6), with pristine TpPa displaying *a* = *b* = 22.7765 Å, *c* = 3.4587 Å, and MBH@TpPa showing *a* = *b* = 22.5471 Å, *c* = 3.5069 Å; all with angles *α* = *β* = 90°, *γ* = 120°. The observed contraction of the *a*-axis and expansion of the *c*-axis upon enzyme incorporation indicates a minor structural adjustment of the framework while maintaining long-range order (Fig. 2a, b, Tables S1 and S2).

The porosity and surface areas of pristine TpPa and MBH@TpPa were evaluated by nitrogen sorption measurements at 77 K after vacuum activation at room temperature. Pristine TpPa exhibited a Brunauer–Emmett–Teller (BET) surface area of 376 m^2^ g^−1^ and a total pore volume of 0.277 cm^3^ g^−1^, while MBH@TpPa showed reduced values of 283 m^2^ g^−1^ and 0.193 cm^3^ g^−1^, respectively, indicating partial blockage of the porous network upon enzyme incorporation (Fig. 2c and S1). Pore size distributions obtained *via* quenched solid density functional theory (QSDFT) revealed sharp, unimodal profiles. The pristine COF displayed a maximum at 1.75 nm, consistent with the theoretical pore diameter of 1.8 nm from structural modeling. Upon MBH incorporation, this maximum shifted to 1.55 nm. This reduction, along with the lower surface area and pore volume, points to partial occupation or blockage of the pore network. Since the MBH has a diameter of around 8 nm and is therefore much larger than an individual COF pore, it is impossible to be encapsulated in a single channel. However, a reduction in surface area may also be attributed to the enzyme residing in cavities formed during the growth of the surrounding COF, where it partially obstructs pore accessibility and may partially extend into the microporous network. Additionally, SEM images suggest that the COF does not contain larger pores or cavities but consists of solid, aggregated particles (Fig. S2).

Thermal stability of the COF framework, assessed *via* thermogravimetric analysis (TGA) under a nitrogen atmosphere, showed negligible mass loss up to 400 °C, highlighting its excellent thermal robustness (Fig. S3).

Fourier-transform infrared (FTIR) spectroscopy revealed characteristic vibrational bands at 1233 cm^−1^ (C–N stretching) and 1575 cm^−1^ (C=C stretching), consistent with the formation of β-ketoenamine linkages (Fig. S4). These findings were corroborated by solid-state ^13^C-NMR spectroscopy, which exhibited a distinct resonance at 184 ppm, attributable to the carbonyl carbon (C=O) of the ketoenamine motif (Fig. S5). Notably, no clear spectroscopic signatures corresponding to the MBH adduct were observed in either FTIR or ^13^C-NMR spectra. This absence is likely due to the low concentration of MBH molecules incorporated within the COF structure, which may be below the detection limits of these techniques. To gain more insights into structural changes occurring during this process, surface enhanced infrared spectroscopy (SEIRAS) complemented by IR-ATR measurements was employed to monitor the different steps of the MBH@TpPa COF formation (Fig. S6), yielding spectral indications of the encapsulation of MBH. Since MBH comprises metal cofactors (the [NiFe] site and 3 Fe–S clusters),^5^*i.e.*, one Ni and twelve Fe atoms per enzyme molecule, this metal content was used as a proxy, to determine the amount of encapsulated MBH, by employing ICP-MS-based metal quantification. The metal contents of pristine TpPa COF and MBH@TpPa COF are summarized in Table S3 and S4. In relation to the original amount of enzyme that was used for the MBH@TpPa preparation, the yield of encapsulated MBH was 41.8 ± 2.1% which is remarkable, as the encapsulation procedure was carried out with a highly diluted (3 µM) MBH solution. The ratio of Fe : Ni in MBH@TpPa was found to be 10.3 : 1 which is close to the theoretical ratio of 12 : 1, based on protein structure determination. This is in close agreement with the experimentally determined Fe : Ni ratio of 11.7 : 1 for the pure enzyme prior to COF encapsulation. Small amounts of Fe and Ni from unknown sources were also detected in the pristine COF. However, because these contributions are much lower and correspond to a Fe : Ni ratio of 24.1 : 1, it can be reasonably concluded that the metals detected in the MBH@TpPa COF predominantly originate from the encapsulated MBH molecules.

Furthermore, electron paramagnetic (EPR) spectroscopy was used to verify the successful incorporation and preserved integrity of the MBH in TpPa COF, based on the characteristic signals from the metal cofactors embedded in the enzyme (Fig. 2d). The typical complex spectroscopic signature between *g* = 2.5 and *g* = 1.8 originates from the paramagnetic [NiFe] center and two Fe–S clusters, which are magnetically coupled.^36,37^ While the pristine TpPa COF is largely EPR-silent, apart from a sharp signal stemming from an organic radical, the MBH@TpPa COF exhibits a spectroscopic pattern very similar to that of MBH in solution, confirming the integration of native-like MBH into the TpPa COF.

### Photocatalytic hydrogen evolution reaction of COFs and hydrogenase-COF hybrids

To investigate the optical and electronic properties of pristine TpPa and MBH@TpPa, we employed solid-state UV-visible diffuse reflectance spectroscopy, valence band (VB) X-ray photoelectron spectroscopy (XPS), and EPR spectroscopy. Both materials exhibited broad visible absorption, with MBH@TpPa showing a slight red shift in the absorption edge (∼675 nm *vs.* ∼658 nm) (Fig. S7). Tauc plot analysis revealed nearly identical optical band gaps of 2.83 eV and 2.82 eV for pristine and MBH-functionalized COFs, respectively (Fig. S8). These band gaps are larger than those reported for conventionally synthesized TpPa COFs, which have been shown to be active photocatalysts for sacrificial H_2_ evolution reaction (HER).^38^ This increase can be attributed to the much milder, aqueous-phase synthesis, which, however, limits the development of extended π-conjugation within the framework. Nevertheless, valence band XPS measurements and the derived band positions indicate that both materials should remain suitable for photocatalytic proton reduction (2H^+^ + 2e^−^→ H_2_) (Fig. S9 and S10).

**Fig. 3:**
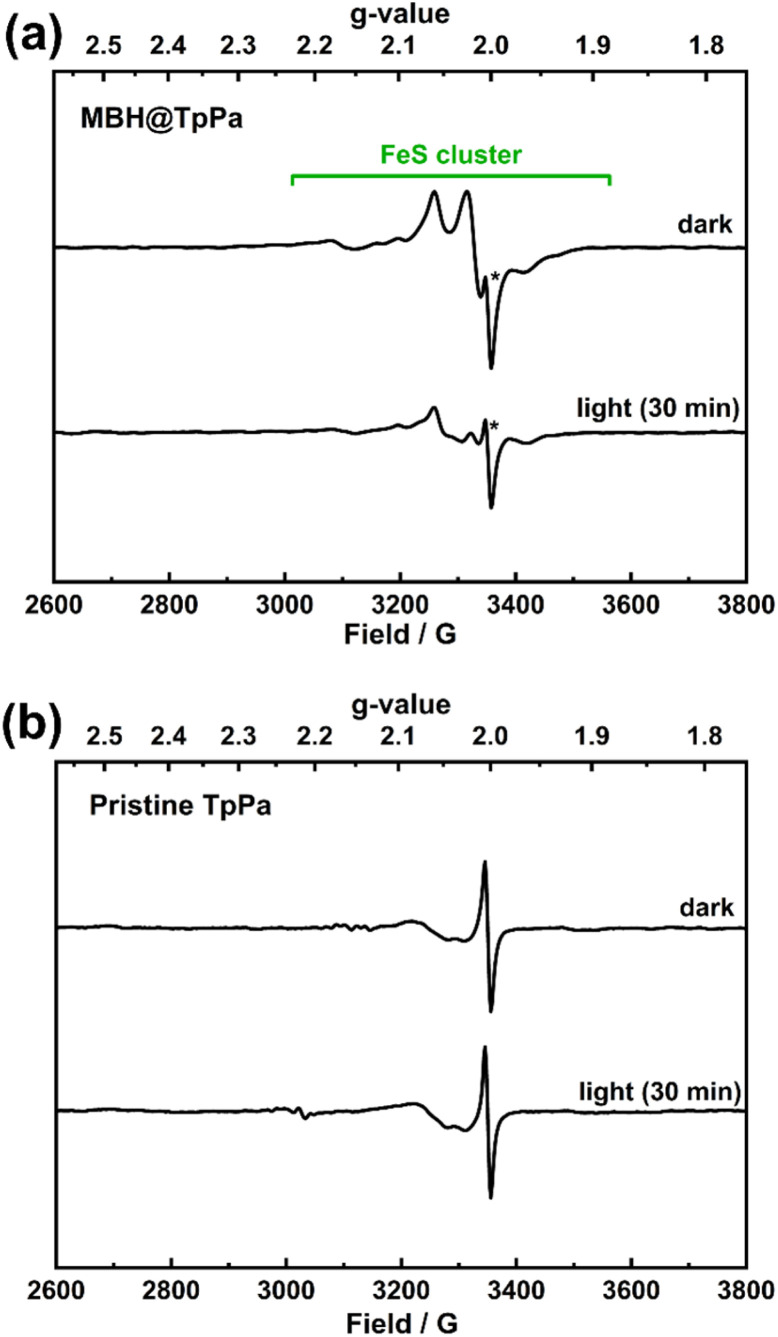
EPR spectra of (a) MBH@TpPa composite recorded at 20 K. (b) Corresponding EPR spectrum of the pristine TpPa COF recorded under the same conditions. The asterisk denotes a sharp signal attributed to an organic radical species inherent to the TpPa COF framework.

The fast electron transfer from the COF backbone to the Fe–S cluster chain of MBH after photo-induced charge separation is a crucial prerequisite for efficient H_2_ production of MBH@TpPa. Utilizing EPR spectroscopy we observed a drastic decrease of the EPR signals from the oxidized paramagnetic metal clusters in MBH after 30 min illumination at room temperature, while the pristine TpPa COF remained unchanged (Fig. 3a and b). This enabled us to directly monitor the almost complete reduction of these cofactors, which supply the electrons necessary for effective proton reduction at the [NiFe] center.

To gain deeper insight into charge separation efficiency, transient photocurrent response measurements were performed under dark and visible-light irradiation for both pristine TpPa and MBH@TpPa COFs. Upon illumination, both materials exhibited increased photocurrent, with MBH@TpPa showing a markedly higher photocurrent density compared to pristine TpPa (Fig. S11). This enhancement indicates more efficient separation of photogenerated charge carriers in the MBH-functionalized COF. The improved photocurrent response can be rationalized by considering the role of MBH as an effective electron acceptor: after photoexcitation of the COF, electrons are rapidly transferred *via* the Fe–S clusters to the [NiFe] active site.

This electron transfer suppresses charge recombination within the COF framework, thereby prolonging charge carrier lifetimes and boosting photocurrent. Such interfacial charge transfer between the COF and enzyme is critical for efficient photocatalytic activity and explains the superior photoelectronic behavior of MBH@TpPa. This observation is in line with the corresponding EPR analyses at room temperature that facilitate to monitor exclusively the organic radical signals (Fig. S12). Upon illumination a significant and reversible increase of an isotropic signal (*g* = 2.004), likely related to organic radicals photogenerated due to charge separation, is observed. The relative increase of this species is clearly higher (approx. a factor of 2) for MBH@TpPa compared to TpPa COF, in line with the trend determined in the above-described findings.

**Fig. 4:**
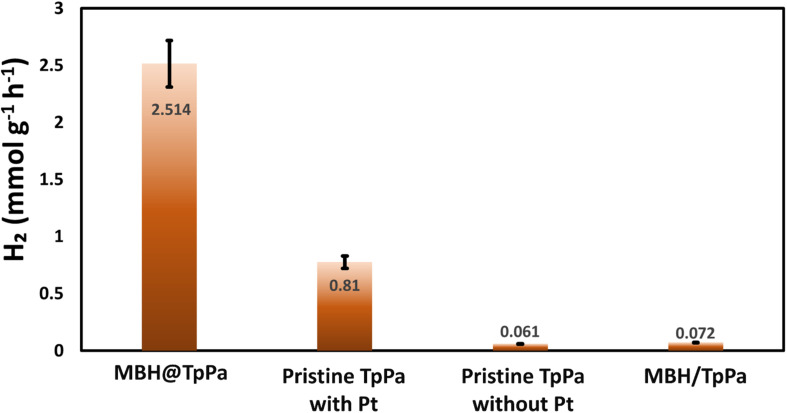
Comparison of the hydrogen evolution rate (HER) for the MBH@TpPa biohybrid, pristine TpPa COF loaded with platinum, pristine TpPa COF without platinum, and a physical mixture of MBH with TpPa COF (MBH/TpPa). Error bars represent the standard deviation from five independent experimental replicates (*n* = 5).

Subsequently, both COF materials were evaluated for their photocatalytic activity toward the HER under visible light irradiation (*λ* > 420 nm). The photocatalytic experiments were performed in aqueous media using 0.1 M ascorbic acid as a sacrificial electron donor, with the solution pH adjusted to 5.5 through the addition of sodium ascorbate (Fig. S13 and S14). Detailed experimental procedures are provided in the SI. The MBH@TpPa COF exhibited markedly enhanced photocatalytic performance, achieving a hydrogen evolution rate of 2.59 mmol g^−1^ h^−1^ in the absence of platinum, significantly surpassing that of the pristine TpPa COF with Pt (0.81 mmol g^−1^ h^−1^) and demonstrating an approximate 43-fold increase relative to the pristine TpPa COF without Pt (Fig. 4). Furthermore, MBH@TpPa COF exhibited performance comparable to previously reported materials (Table S5). These results highlight the synergistic effect between the enzyme active sites and the COF scaffold.

To rationalize the enhanced photocatalytic H_2_ evolution by MBH@TpPa (Fig. S15), we propose a mechanistic model based on interfacial electron transfer guided by the experimentally determined band structures (Fig. S10). The conduction band (CB) of the MBH@TpPa composite is positioned at approximately −3.40 eV *vs.* vacuum (−1.04 V *vs.* SHE), which is more negative and thus more reductive than that of pristine TpPa (−3.69 eV *vs.* vacuum, −0.75 V *vs.* SHE). This elevated energy level provides a thermodynamically favorable offset for electron injection into the enzyme. Critically, the CB of MBH@TpPa is significantly more negative than the catalytic potential for the H^+^/H_2_ couple (*E*°′ ≈ −0.414 V *vs.* SHE) at the [NiFe]-hydrogenase active site, resulting in a substantial driving force (Δ*E* > 0.6 eV) for electron transfer from the photosensitizer to the enzyme.^39,40^ Under visible-light irradiation, TpPa absorbs photons to generate electron–hole pairs; photogenerated electrons in its CB are injected into MBH and relayed *via* Fe–S clusters to the [NiFe] catalytic center, where proton reduction occurs (2H^+^ + 2e^−^ → H_2_). Concurrently, holes in the valence band oxidize ascorbic acid, serving as sacrificial donor and suppressing recombination. This directional charge transfer, together with spatial separation of charge carriers, is consistent with the enhanced photocurrent and EPR signatures observed for MBH@TpPa and directly enables its exceptional activity.

Control experiments were conducted to elucidate the role of MBH incorporation. Isolated MBH, when tested independently in the absence of TpPa COF, showed negligible catalytic activity due to the absence of a photosensitizer. Moreover, a physical mixture of MBH and TpPa COF (denoted MBH/TpPa) exhibited substantially lower photocatalytic activity (0.072 mmol g^−1^ h^−1^). This observation is crucial: if the enhanced activity of the MBH@TpPa composite were simply due to unspecific MBH binding to TpPa COF or leached metal species from denatured enzyme, the physical mixture would display a similar enhancement. The pronounced difference in activity instead confirms that the high performance relies on the intimate integration and structural integrity of the enzyme within the COF framework, achieved through *in situ* encapsulation. Additional experiments performed in the absence of light confirmed no detectable hydrogen evolution, verifying the photocatalytic nature of the process (Fig. S16). Importantly, the MBH@TpPa COF demonstrated stable H_2_ production over a continuous 6 hours irradiation period, indicating the protective role of the COF scaffold in preserving MBH activity (Fig. S17). The apparent quantum efficiency (AQE) at 420 nm was determined to be 0.27%, further corroborating the photocatalytic efficacy of the hybrid material. While increasing the synthesis time to 1 h enhances the crystallinity of the COF, a slight decrease in catalytic activity shows that crystallinity of the framework alone does not control optimal activity (Fig. S18).

**Fig. 5:**
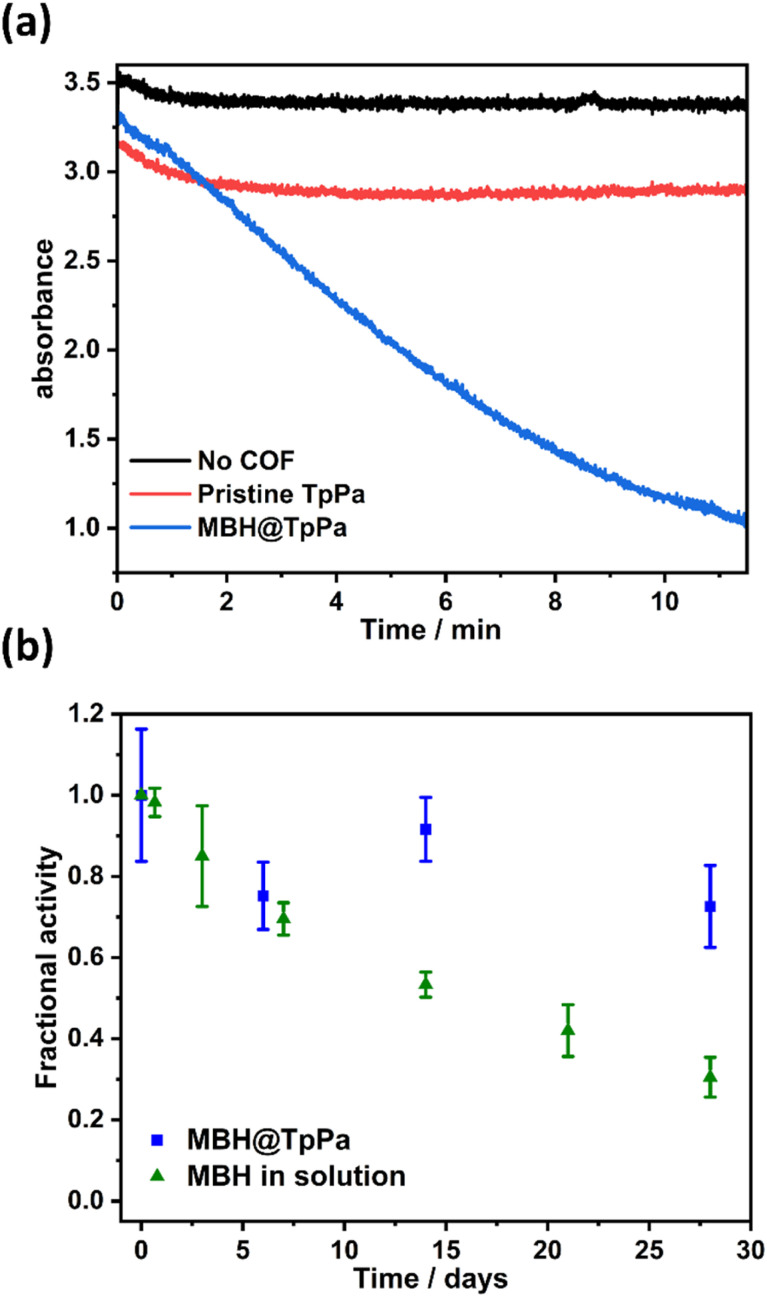
(a) Hydrogen oxidation activity, monitored by the time-dependent reduction of the artificial electron acceptor methylene blue. Representative traces are shown for the MBH@TpPa composite, the pristine TpPa COF, and a control containing no COF. (b) Long-term operational stability. The retained catalytic activity of MBH encapsulated within the TpPa COF is compared to that of free MBH in solution over a period of 28 days.

In order to verify that the enzyme is still active after encapsulation, pristine TpPa COF as well as MBH@TpPa COF was subjected to H_2_ oxidation activity assays (Fig. 5a and S19).^41^ In case of no added COF as well as the addition of pristine TpPa COF a short decrease in absorbance was observed, which is presumably due to thermal equilibration of the redox dye in the photometer, followed by a stable signal indicating no H_2_ oxidation activity. In case of MBH@TpPa COF a clear decrease of absorbance over time was observed which is caused by the reduction of methylene blue, derived from H_2_ oxidation by MBH. However, the estimated turnover frequency dropped from 217 s^−1^ of MBH in solution (taking into account the activity losses in the course of the MBH@TpPa COF preparation, *i.e.*, imidazole buffer and EtOH precipitation) to 13 s^−1^ of encapsulated MBH. This is likely related to the diffusion-limited interaction of the redox dye methylene blue with the MBH@TpPa, proving that the MBH is indeed deeply encapsulated within the COF scaffold. Presumably, a certain activity decrease can also be ascribed to a fraction of inactivated enzyme. Nevertheless, our data clearly demonstrate that MBH was still active in H_2_ oxidation after incorporation into the COF material and fully reduced the provided redox mediator.

Next, the stability of encapsulated MBH *vs.* MBH in solution was tested by storing MBH@TpPa COF and MBH in solution at 4 °C for 28 days. In the meantime, samples were drawn for activity determination (Fig. 5b). The fractional activity of MBH in solution dropped to 30.5%, while MBH@TpPa COF retained 72.6% of its initial activity, demonstrating a significant stabilization of encapsulated MBH *versus* MBH in solution.

In order to test the stability of encapsulation, we performed leaching tests (see SI for details). To do so, MBH@TpPa COF was stirred over night in buffer and the supernatant was analyzed for enzyme activity. In parallel, the same amount of MBH@TpPa COF was washed 5 times with buffer using a 0.2 µm filter, and the filtrate was submitted to enzyme activity assays. In order to make sure that even the slightest enzyme activity would be detected, the filtrate and the stirred fraction supernatant were each concentrated. In both cases no enzyme activity was detected. This indicated that there was no detectable leaching from the MBH@TpPa COF composite. As an ultimate test, MBH@TpPa COF was mixed with SDS-PAGE sample buffer and heated to 95 °C for 2 minutes to extract the protein for visualization *via* SDS-PAGE (Fig. S21). Since this method fully unfolds proteins, any merely associated protein would detach from the COF easily. Controls showed the typical two bands corresponding to the large and small subunits of MBH, while, in the lane loaded with MBH@TpPa COF, no protein became visible even though theoretically more protein compared to the control lanes was loaded. As additional controls, macroporous COF loaded with MBH after COF synthesis,^28^ was treated in the same way and here clearly the bands corresponding to MBH were visible. This demonstrates that MBH is either covalently attached to the COF or is so deeply embedded within the matrix that even the denatured protein cannot escape, in either case, the interaction is far stronger than simple adsorption or physical trapping.

## Conclusions

This study demonstrates an advanced strategy for the direct integration of [NiFe]-hydrogenase into TpPa COF *via* a rapid *in situ* encapsulation, avoiding high temperatures and harsh organic solvents, yielding a biohybrid material (MBH@TpPa) with outstanding bidirectional catalytic activity. The composite exhibits significantly enhanced hydrogen evolution rates, outperforming both Pt-loaded and Pt-free COFs, and maintains long-term stability in H_2_ oxidation. These results highlight the strong electronic coupling between the enzyme's active site and the COF scaffold, which facilitates efficient charge transfer and supports the system's superior performance. More broadly, the inherent photoactivity of COFs enables the delivery of low-potential, photoexcited electrons to drive demanding, enzyme-controlled redox reactions. This strategy offers a versatile, noble metal-free platform for sustainable photocatalysis and may be extended to other redox enzymes for light-driven, energetically uphill transformations. Furthermore, the H_2_ oxidation activity of MBH@TpPa can be leveraged for atom-efficient hydrogenation reactions,^42^ for example, by co-encapsulating complementary enzymes to establish robust COF-protected cascade systems for the production of value-added compounds.

## Author contributions

I. E. K. designed and investigated the project, synthesized the materials, interpreted the analytical data, performed the photocatalytic HER experiments, and wrote the original draft. S. F. synthesized and characterized the enzyme, performed the hydrogen oxidation experiments, and contributed to writing the original draft. P. D. performed the XRD powder refinement and structural analysis. C. L. and C. T. performed the EPR measurements. A. F. T. and I. Z. performed the SEIRAS and ATR-IR measurements. A. A. O. and O. L. revised the manuscript. A. T. designed and supervised the project and contributed to writing the original draft. All authors have given approval to the final version of the manuscript.

## Conflicts of interest

There are no conflicts to declare.

## Supplementary Material

SC-017-D6SC01349J-s001

## Data Availability

The data supporting this article have been included as part of the supplementary information (SI). Supplementary information is available. See DOI: https://doi.org/10.1039/d6sc01349j.

## References

[cit1] Lubitz W., Ogata H., Rüdiger O., Reijerse E. (2014). Chem. Rev..

[cit2] Goldet G., Brandmayr C., Stripp S. T., Happe T., Cavazza C., Fontecilla-Camps J. C., Armstrong F. A. (2009). J. Am. Chem. Soc..

[cit3] Goldet G., Wait A. F., Cracknell J. A., Vincent K. A., Ludwig M., Lenz O., Friedrich B., Armstrong F. A. (2008). J. Am. Chem. Soc..

[cit4] Lenz O. , Lauterbach L., Frielingsdorf S. and Friedrich B., in Biohydrogen, DE GRUYTER, 2015, pp. 61–96

[cit5] Fritsch J., Scheerer P., Frielingsdorf S., Kroschinsky S., Friedrich B., Lenz O., Spahn C. M. T. (2011). Nature.

[cit6] Reisner E., Powell D. J., Cavazza C., Fontecilla-Camps J. C., Armstrong F. A. (2009). J. Am. Chem. Soc..

[cit7] Caputo C. A., Gross M. A., Lau V. W., Cavazza C., Lotsch B. V., Reisner E. (2014). Angew. Chem., Int. Ed..

[cit8] Caputo C. A., Wang L., Beranek R., Reisner E. (2015). Chem. Sci..

[cit9] Wang X., Lan P. C., Ma S. (2020). ACS Cent. Sci..

[cit10] Li P., Chen Q., Wang T. C., Vermeulen N. A., Mehdi B. L., Dohnalkova A., Browning N. D., Shen D., Anderson R., Gómez-Gualdrón D. A., Cetin F. M., Jagiello J., Asiri A. M., Stoddart J. F., Farha O. K. (2018). Chem.

[cit11] Lyu F., Zhang Y., Zare R. N., Ge J., Liu Z. (2014). Nano Lett..

[cit12] Liang K., Ricco R., Doherty C. M., Styles M. J., Bell S., Kirby N., Mudie S., Haylock D., Hill A. J., Doonan C. J., Falcaro P. (2015). Nat. Commun..

[cit13] Liang J., Liang K. (2020). Adv. Funct. Mater..

[cit14] Liang W., Carraro F., Solomon M. B., Bell S. G., Amenitsch H., Sumby C. J., White N. G., Falcaro P., Doonan C. J. (2019). J. Am. Chem. Soc..

[cit15] Tang Z., Li X., Tong L., Yang H., Wu J., Zhang X., Song T., Huang S., Zhu F., Chen G., Ouyang G. (2021). Angew. Chem., Int. Ed..

[cit16] Wied P., Carraro F., Bolivar J. M., Doonan C. J., Falcaro P., Nidetzky B. (2022). Angew. Chem., Int. Ed..

[cit17] Lin R. B., Chen B. (2022). Chem.

[cit18] Liu S., Sun Y. (2023). Angew. Chem., Int. Ed..

[cit19] Sun Q., Aguila B., Lan P. C., Ma S. (2019). Adv. Mater..

[cit20] Kandambeth S., Venkatesh V., Shinde D. B., Kumari S., Halder A., Verma S., Banerjee R. (2015). Nat. Commun..

[cit21] Zhu Q., Zheng Y., Zhang Z., Chen Y. (2023). Nat. Protoc..

[cit22] Khalil I. E., Das P., Küçükkeçeci H., Dippold V., Rabeah J., Tahir W., Roeser J., Schmidt J., Thomas A. (2024). Chem. Mater..

[cit23] Khalil I. E., Das P., Thomas A. (2024). Acc. Chem. Res..

[cit24] Paul S., Gupta M., Dey K., Mahato A. K., Bag S., Torris A., Gowd E. B., Sajid H., Addicoat M. A., Datta S., Banerjee R. (2023). Chem. Sci..

[cit25] Li M., Qiao S., Zheng Y., Andaloussi Y. H., Li X., Zhang Z., Li A., Cheng P., Ma S., Chen Y. (2020). J. Am. Chem. Soc..

[cit26] Sun Q., Fu C. W., Aguila B., Perman J., Wang S., Huang H. Y., Xiao F. S., Ma S. (2018). J. Am. Chem. Soc..

[cit27] Paul S., Gupta M., Kumar Mahato A., Karak S., Basak A., Datta S., Banerjee R. (2024). J. Am. Chem. Soc..

[cit28] Khalil I. E., Waffo A. F. T., Das P., Katz S., Kunow S., Lorent C., Ziouani Y., Tahir W., Owusu A. A., Lenz O., Zebger I., Frielingsdorf S., Thomas A. (2025). Adv. Funct. Mater..

[cit29] Wu Z., Shan H., Jiao Y., Huang S., Wang X., Liang K., Shi J. (2022). Chem. Eng. J..

[cit30] Zheng Y., Zhang S., Guo J., Shi R., Yu J., Li K., Li N., Zhang Z., Chen Y. (2022). Angew. Chem., Int. Ed..

[cit31] Chao H., Zhou Z., He W., Li M., Yuan X., Su P., Song J., Yang Y. (2022). ACS Appl. Mater. Interfaces.

[cit32] Talekar S., Kim Y., Wee Y., Kim J. (2023). Chem. Eng. J..

[cit33] Liang J., Ruan J., Njegic B., Rawal A., Scott J., Xu J., Boyer C., Liang K. (2023). Angew. Chem., Int. Ed..

[cit34] Hao L., Zhu Q., Qiao X., Shi Q., Liu Y., Wang T., Lin E., Cheng P., Zhang Z., Chen Y. (2025). Angew. Chem., Int. Ed..

[cit35] Guo L., Zhang Y., Yu Z., Krishna R., Luo F. (2023). Chem. Mater..

[cit36] Saggu M., Zebger I., Ludwig M., Lenz O., Friedrich B., Hildebrandt P., Lendzian F. (2009). J. Biol. Chem..

[cit37] Pandelia M. E., Nitschke W., Infossi P., Giudici-Orticoni M. T., Bill E., Lubitz W. (2011). Proc. Natl. Acad. Sci. U. S. A.

[cit38] Dong P., Wang Y., Zhang A., Cheng T., Xi X., Zhang J. (2021). ACS Catal..

[cit39] Panich J., Fong B., Singer S. W. (2021). Trends Biotechnol..

[cit40] Shafaat H. S., Rüdiger O., Ogata H., Lubitz W. (2013). Biochim. Biophys. Acta Bioenerg..

[cit41] Lenz O., Lauterbach L., Frielingsdorf S. (2018). Methods Enzymol..

[cit42] Sokolova D., Vincent K. A. (2024). Chem. Commun..

