# Supplementary material for: Rapid in situ encapsulation of [NiFe]-hydrogenase into covalent organic frameworks for robust hydrogen oxidation and evolution
Source: Chem Sci. 2026 May 25;17(28):14020–8. doi: 10.1039/d6sc01349j (PMC13255557; doi:10.1039/d6sc01349j)
Supplement: SC-017-D6SC01349J-s001 [file SC-017-D6SC01349J-s001.pdf]

Supplementary Information (SI) for Chemical Science.
This journal is © The Royal Society of Chemistry 2026

Rapid in situ encapsulation of [NiFe]-hydrogenase into covalent organic frameworks for robust hydrogen oxidation and evolution.

Islam E. Khalil[‡,a] Stefan Frielingsdorf[‡,b] Prasenjit Das,[a] Christian Lorent,[b] Christian Teutloff,[c] Augustine A. Owusu,[a] Armel F. Tadjoung Waffo,[b] Ingo Zebger,[b] Oliver Lenz,[b] Arne Thomas*[a, d]

(a) Department of Chemistry, Functional Materials Technische Universität Berlin 10623 Berlin, Germany

(b) Department of Chemistry, Biophysical Chemistry, Technische Universität Berlin, Straße des 17. Juni 135, 10623 Berlin, Germany

(c) Department of Physics, Freie Universität Berlin, Arnimallee 14, 14195 Berlin, Germany

(d) Department of Chemistry, Technische Universität München, Lichtenbergstr, 4, 85748 Garching

Materials and Methods

1.1. Materials

All chemicals were purchased from commercial sources and used without further treatment: phloroglucinol (Acros Organics, 99+%), hexamethylenetetramine (Sigma Aldrich, ACS reagent, ≥ 99%), trifluoroacetic acid (Carl Roth, ≥ 99.9%), hydrochloric acid (Carl Roth, 37%), 1,4-Phenylendiamin (Sigma Aldrich), Imidazol (Sigma Aldrich), 32.5% of ultrapure Nitric acid (Carl Roth), Ethanol (Carl Roth, ≥ 99%), Potassium dihydrogen phosphate and dipotassium hydrogen phosphate were purchased from Fischer Chemicals, Acetic acid, Hexachloroplatinic acid (Sigma Aldrich), Ascorbic acid (Alfa Aesar, 98.5%), Sodium ascorbate (sigma Aldrich), Sodium sulfate (Alfa Aesar, 98.5%), Sodium chloride (BLDpharm 99.9%).

1.2. Characterization

X-ray Powder Diffraction patterns were collected on a Bruker D8 Advance diffractometer in reflection geometry operating with a Cu Kα anode (λ = 1.54178 Å) operating at 40 kV and 40 mA. Samples were ground and mounted as loose powders onto a Si sample holder. PXRD patterns were collected from 2 to 60 2θ degrees with a step size of 0.02 degrees and an exposure time of 2 seconds per step.

Nitrogen Sorption Measurements were performed at 77 K using an Autosorb-iQ-MP from Quantachrome. Prior to the analysis the samples were dried and degassed at room temperature and 120 °C for 12 h.

Fourier transform infrared spectroscopy (FTIR) analyses were carried on Varian 640IR spectrometer equipped with an ATR cell.

Thermogravimetric analyses (TGA) were performed using a TGA Q500 thermal analysis system under a N_2 atmosphere from room temperature to 800 °C at a ramping rate of 5 °C /min.

X-Ray Photoelectron Spectroscopy (XPS) The X-ray photoelectron spectroscopy measurements were performed on a Thermo Scientific K-Alpha+ X-ray Photoelectron Spectrometer System with a hemispheric 180° dual-focus analyser and a 128-channel detector. The X-ray monochromator used micro-focused Al-Kα radiation.

Solid-state diffuse reflectance Ultraviolet–Visible spectroscopy (UV-Vis) spectra of the COFs have been collected on Varian Cary 300 UV-Vis Spectrophotometer.

Electron paramagnetic resonance spectroscopy (EPR) was either done on a Brucker EMX plus X-Band spectrometer equipped with an ER 4122 super-high Q (SHQE) resonator (Bruker Corporation), an Oxford ESR900 helium flow cryostat (Oxford Instruments) and an ITC4 temperature controller (Oxford Instruments) or a laboratory-built X-Band spectrometer equipped with a SHQ resonator resonator (Bruker Corporation), an ESR 910 helium flow cryostat and an ITC503 temperature controller (Oxford Instruments). Baseline correction was performed by the subtraction of a reference spectrum obtained from a sample of buffer

solution or the empty resonator recorded with the same experimental parameters. For subsequent corrections a polynomial or spline function was used. Experimental parameters used were: 1 mW microwave power, microwave frequency 9.3 respectively 9.4 GHz, modulation amplitude 10 G and 100 kHz modulation frequency.

2. Experimental section

2.1. Synthesis of triformylphloroglucinol (Tp)[1]

Phloroglucinol (6.0 g, 49 mmol), hexamethylenetetramine (HMTA, 15.1 g, 108 mmol) and trifluoroacetic acid (TFA, 90 mL) were refluxed at 100 °C under N_2 for 2.5 h. After that, 150 mL of 3 M HCl was added slowly and the mixture was heated at 100 °C for 1 h. After cooling to room temperature, the solution was filtered through Celite, extracted with 350 mL dichloromethane and the solution was evaporated under reduced pressure to yield 1.6 g of an off-white powder. ^{1}H NMR indicated near 99% purity; a pure sample was obtained by sublimation.

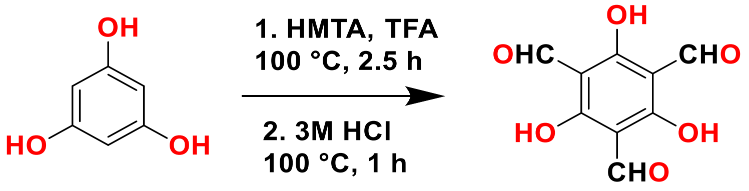

Scheme S1. *Structure of the monomer triformylphloroglucinol (Tp).*

2.2. Synthesis of Pristine TpPa COF

Following some modifications to a reported method,[2] a mixture of p-phenylenediamine (PDA, 4.9 mg, 0.045 mmol), TP (6.3 mg, 0.03 mmol), and imidazole (20 mg, 0.3 mmol) was dissolved in 0.5 mL of water and stirred for 5 minutes. Then, an additional 0.5 mL of water and 4 µL of 3 M acetic acid (AcOH) were added, and the mixture was stirred for another 5 minutes until a clear solution formed. To this solution, 0.3 mL of ethanol (EtOH), miscible with water and capable of hydrogen bonding, was added as a precipitant, immediately inducing the rapid and high-quality formation of a precipitate. The resulting solid was collected by filtration and thoroughly washed with water.

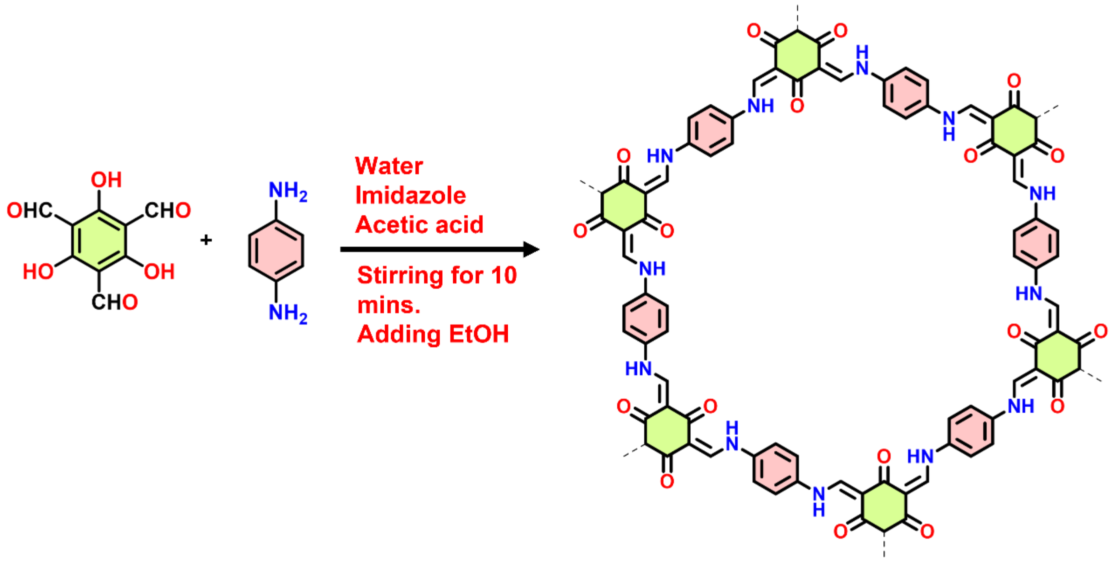

Scheme S2*. Chemical structure of the pristine TpPa covalent organic framework.*

Table S1. Crystallographic data for MBH@TpPa. Fractional atomic coordinates for the refined structural model of the MBH@TpPa biohybrid composite.

**MBH@TpPa _Pawley**				
Space Group: *P-6*<br>a = 22.5471 Å, b = 22.5471 Å, c = 3.5069 Å<br>$\alpha = \beta$ = 90.00°, γ = 120.00°<br>R_p: 2.47 % and R_{wp}: 3.31 %				
**Atom label**	**Atom type**	***x***	***y***	***z***
C1	C	-0.56977	-0.54512	1
C2	C	-0.52468	-0.56982	1
C3	C	-0.45444	-0.52465	1
C4	C	-0.43023	-0.45488	1
C5	C	-0.47531	-0.43017	1
C6	C	-0.54556	-0.47535	1
H7	H	-0.62376	-0.5804	1
H8	H	-0.54479	-0.62402	1
H9	H	-0.37624	-0.4196	1
H10	H	-0.45521	-0.37598	1
C11	N	-0.40671	-0.54805	1
C12	N	-0.59329	-0.45195	1
C13	C	0.59268	0.28638	0
C14	C	0.63969	0.25896	0
N15	C	0.57438	0.38202	0
O16	O	0.52884	0.24639	0
C17	C	0.25896	0.61927	0
C18	C	0.3063	0.59268	0
O19	O	0.24639	0.71756	0

N20	C	0.19236	0.57438	0

Table S2. Crystallographic data for pristine TpPa COF. Fractional atomic coordinates for the refined structural model of the pristine TpPa covalent organic framework.

**Pristine TpPa_Pawley**				
Space Group: *P-6*<br>a = 22.7765 Å, b = 22.7765 Å, c = 3.4587 Å<br>$\alpha = \beta$ = 90.00°, γ = 120.00°<br>R_p: 2.58 % and R_{wp}: 3.30 %				
**Atom label**	**Atom type**	***x***	***y***	***z***
C1	C	-0.56977	-0.54512	1
C2	C	-0.52468	-0.56982	1
C3	C	-0.45444	-0.52465	1
C4	C	-0.43023	-0.45488	1
C5	C	-0.47531	-0.43017	1
C6	C	-0.54556	-0.47535	1
H7	H	-0.62376	-0.5804	1
H8	H	-0.54479	-0.62402	1
H9	H	-0.37624	-0.4196	1
H10	H	-0.45521	-0.37598	1
C11	N	-0.40671	-0.54805	1
C12	N	-0.59329	-0.45195	1
C13	C	0.59268	0.28638	0
C14	C	0.63969	0.25896	0
N15	C	0.57438	0.38202	0
O16	O	0.52884	0.24639	0
C17	C	0.25896	0.61927	0

C18	C	0.3063	0.59268	0
O19	O	0.24639	0.71756	0
N20	C	0.19236	0.57438	0

2.3. Enzyme preparation

The membrane-bound hydrogenase from *Cupriavidus necator* was prepared following published protocols.[3] In order to prevent introduction of additional compounds to the following COF synthesis step, the buffer of the MBH sample was exchanged to 150 mM imidazole pH 6.0 using a PD MiniTrap® G-25 (Cytiva) column. The sample was subsequently concentrated to 100-150 µM protein concentration using ultrafiltration (VIVASPIN 500, 50,000 MWCO PES, Sartorius). In an initial trial, the buffer was exchanged using a Superdex 200 Increase 10/300 GL column on an Äkta pure™ system **(Fig. S19).** The specific activity dropped by 19.2 % upon buffer exchange.

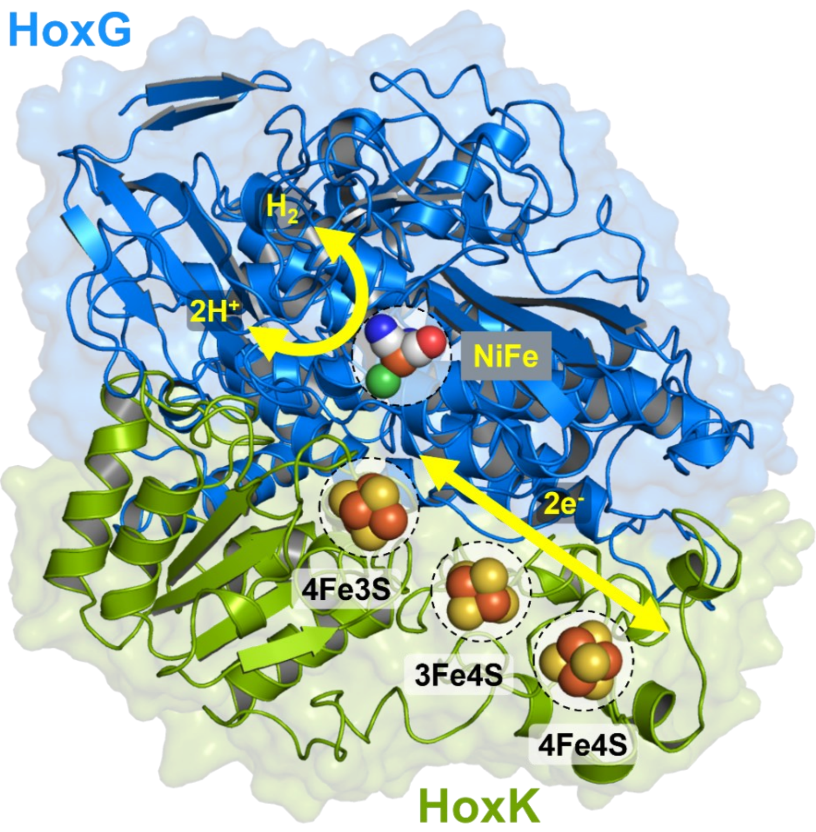

Scheme S3. *Schematic structure of the membrane-bound hydrogenase (MBH) from Cupriavidus necator. The model highlights the [NiFe] active site and electron-transfer Fe-S clusters based on structural data.*[4,5]

2.4. Synthesis of MBH@TpPa

A mixture of p-phenylenediamine (4.9 mg, 0.045 mmol), TP (6.3 mg, 0.03 mmol), and imidazole (20 mg, 0.3 mmol) was dissolved in 0.5 mL of water and stirred for 5 minutes. Subsequently, 20 µL of 100-150 µM MBH solution was added with moderate stirring for 2-3

minutes. An additional 0.5 mL of water and 4 µL of 3 M acetic acid (AcOH) were then added, and the resulting mixture was stirred for another 5 minutes until a clear solution was obtained. To this clear solution, 0.3 mL of ethanol (EtOH), miscible with water and capable of hydrogen bonding, was added as a precipitant, immediately inducing the rapid and high-quality formation of a precipitate. The resulting solid was collected by filtration and thoroughly washed with water.

2.5. Surface-enhanced infrared absorption spectroscopy (SEIRAS)

Prior to all measurements, a thin nanostructured gold film was chemically deposited onto a silicon prism applicable for Attenuated Total Reflection (ATR) IR spectroscopy and subsequently cleaned by an electrochemical procedure.[6] The prism was mounted into a custom-built spectroelectrochemical cell and placed in a Bruker IFS66v/s IR spectrometer, equipped with a liquid nitrogen-cooled Mercury-Cadmium-Telluride (MCT) detector. The gold film served as the working electrode, while a platinum wire and an Ag/AgCl electrode were chosen as counter and reference electrodes, respectively. We recorded spectra with a resolution of 4 cm^{-1}, averaging 400 scans for each spectrum. Following the procedure described above, a volume of 0.25 mL water was poured into the SEIRA cell, and reference spectra were recorded. Subsequently, 3.15 mg of TP was added to the solution and 10 spectra were recorded, while a N_2-stream gas was used to mix the solution during the entire measurement. After recording the spectra, 2.45 mg of the second monomer p-phenylenediamine (PDA) was added and further spectra were subsequently recorded. To ensure the encapsulation of MBH during the polymerization process, 50 µL of 150 µM MBH solution was added to the solution before pouring additional 0.25 mL of water containing 10 mg imidazol and 2 µL of acetic acid. The solution was then mixed for 5 minutes using a N_2-stream gas. 0.15 mL of ethanol (EtOH) were subsequently added, resulting in the formation of a precipitate. Spectra related to the addition of each chemical were directly recorded after their insertion. The monomers as well as the resulting MBH@TpPa COF were also measured as powder in the ATR mode for comparison.

2.6. *In situ* formation of the MBH@TpPa COF followed by SEIRAS.

Absorbance spectra of the different steps of the MBH@TpPa COF formation are displayed in **(Fig. S5 a)**. The SEIRA spectrum of the TP in black, displays different bands attributable to aromatic and vinyl ν(C=C) stretching modes (1600–1400 cm^{-1}) as well as δ(C–H) bending modes (1400–1000 cm^{-1}). It should be recalled that the enhancement affect observed in SEIRAS is restricted to 8 nm from the Au-surface (first selection rule). Therefore, the relative low concentration of TP in solution leads to the detection of rather weak absorptions. For a better comparability with other spectra, the spectrum of TP has been multiplied by a factor of 4. Moreover, in SEIRAS only absorptions with dipole moment changes perpendicular are spectroscopically visible (second selection rule).[7] Thus, not all modes could be detected with SEIRAS.

To overcome this limitation, ATR measurement shown in **(Fig. S5 b)** was performed on dried monomers (TP and PDA) as well as on the resulting MBH@TpPa COF for comparison. Addition of PDA leads to the disappearance of the band centered at 1640 cm^{-1} that was attributed to ν(C=O) stretching modes of the aldehyde groups of the TP accompanied by an increase of aromatic and vinyl ν(C=O), ν(C=C) stretching (1600–1400 cm^{-1}) as well as δ(C–H) bending modes (1400–1000 cm^{-1}). These observations suggest the formation of the COF as depicted above in scheme 2. Notably, additional bands were detected, centered at 1673 cm^{-1} and 1552 cm^{-1}, respectively. These bands are located in the region characteristic for the protein

backbone (amide I and II bands)[8] and can tentatively be assigned to encapsulated MBH and are more pronounced in the corresponding difference spectrum after and before adding the enzyme to the reaction solution (3-minus-2).

2.7. Photocatalytic H_2 evolution parameters

All photocatalytic reactions were conducted in a two–neck 32 mL side irradiation quartz reactor having a circulation system to maintain the temperature. 3 mg COF powder was dispersed in Ascorbic acid / Sodium ascorbate/water (16.8mg / 337.6mg/18ml) via sonication. Subsequently, 1.8 µl of H_2PtCl_6 aqueous solution (8wt%) was added in the case of pristine TpPa with Pt, and briefly sonicated and transferred into the reactor. Prior to irradiation, the reactor was sealed with rubber stoppers and degassed by Argon for 15 minutes. After that, the reactor was irradiated with a 300 W Xe lamp (L.O.T–Quantum design) with 420 nm long pass filters. Throughout the reaction, the temperature of the system was maintained at 20°C by water circulating. Manually gas samples were collected from the headspace using gas-tight syringes and H_2 was quantified by gas chromatography (FULI Instruments, GC9790 II(PLF01)) equipped with a thermal conductivity detector. Each photocatalytic HER experiment was performed with five independent replicates (n=5) to ensure reproducibility, and the reported data represent the average values with standard deviations.

2.8. AQY Measurements for HER

The apparent quantum yield (AQY) was determined under 300 W Xe lamp (with monochromatic light at (420 nm) , and the light intensity was measured by a ThorLabs PM100D Power with a photodiode sensor. The AQY was calculated using the following equation:

$$AQY\% = \frac{[\mathrm{H_2\ produced\ (mol)}]\mathrm{x}\ 2}{\mathrm{Photon\ number\ entered\ into\ the\ reactor\ (mol)}}\mathrm{x100}$$

$$= \frac{[N_A\ \mathrm{x\ h\ x\ c}]\ \mathrm{x}\ [\mathrm{H_2\ produced\ (mol)}]\ \mathrm{x}\ 2}{\mathrm{I\ x\ S\ x\ t\ x}\ \lambda}\mathrm{x100}$$

Where, N_A is Avogadro's constant (6.022×10^{23} mol^{-1}), *h* is the Planck constant (6.626×10^{-34} J s), c is the speed of light (3×10^{8} m s^{-1}), *S* is the irradiation area (cm^2), *I* is the intensity of irradiation light (W cm^{-2}), *t* is the photoreaction time (s), λ is the wavelength of the monochromatic light (m).

2.9. Photocurrent Measurement.

Photocurrent measurements are performed with a three-electrode set-up from Metrohm (Autolab PGSTAT302N), using a Pt counter electrode, an Ag/AgCl (3M NaCl) reference electrode and Na_2SO_4 (0.5M in water) as electrolyte. The working electrode was fabricated by

mixing under sonication 5 mg COF with water, ethanol (50 µL each) and 1% (w/v) Nafion solution for 30 mins. Then, 2 µL of the suspension was drop-casted onto 0.25 cm^2 of a Fluorine-doped Tin Oxide (FTO) substrate and dried under ambient conditions. Spare FTO surface was covered with isolating paint. Photocurrent measurements were performed at 1V bias under periodical illumination from the back (40s intervals, visible light, 1 W m^{-2}).

2.10. Metal quantification by ICP-MS

Samples for metal quantification were digested in a final concentration of 32.5 % of ultrapure HNO_3 and a final volume of 1 ml at 90 °C overnight. Samples were filled with ultrapure water to 15 ml total volume prior to quantification by inductively coupled plasma-mass spectrometry.[9]

2.11. H_2 oxidation activity determination

Activity assays were performed as published previously[3] with the following modifications.
A Cary300 spectrophotometer equipped with a magnetic stirrer was used in order to prevent sedimentation of the COF material during measurements. The general principle of the activity assay is depicted in **Fig. S19**.

2.12. Stability of MBH toward EtOH

The stability of MBH toward EtOH was tested as follows: 20 µl of 3.3 µM MBH were mixed with either 6 µl buffer (control) or 6 µl EtOH to reach a final concentration of 23 % EtOH as in the COF precipitation step. The mixtures were incubated for 10 minutes on ice and subsequently 10 µl each were subjected to a previously described activity test[3]. The tests were repeated 6 times for each set (buffer and EtOH, respectively) (n=6).

2.13. Preparation and illumination of the EPR samples

An aliquot of the COF materials as described in 1.2 and 1.4 was transferred to an EPR tube and immediately frozen in liquid nitrogen. Illumination of the EPR samples was done within the resonator using a DCR III 150 W halogen lamp from Polytec. To monitor the formation of radical signals from charge separated species at room temperature the COF materials were dried at 100°C under vacuum for 12 hours to remove the remaining water. To detect the photo-induced reduction of the FeS cluster in MBH@TpPA the sample was first measured at 20 K, warmed up to room temperature, illuminated for 0.5 hours and measured again at 20 K.

2.14. Leaching test

Freshly synthesized MBH@TpPa COF where in total 600 µg MBH were used for the synthesis was resuspended by grinding in a mortar in a total volume of 2 ml MBH buffer (50 mM Potassium phosphate buffer (KPi), 150 mM NaCl, pH 5.5). The sample was split into 4 aliquots of 500 µl each. The first aliquot was used to assay H_2 oxidation activity of the freshly synthesized MBH@TpPa COF sample and the activity observed, was in the usual range. A second aliquot was diluted 1:10 with MBH buffer to a total volume of 5 ml and stirred at 4 °C

for 16 h. The sample was centrifuged at 4696 × g for 20 min at 4 °C. The supernatant was filtered through a 0.2 µm regenerated cellulose syringe filter (low protein binding) and subsequently concentrated by ultrafiltration (50 kDa molecular weight cutoff) to ~200 µl. The sample was analyzed for catalytic H_2 oxidation activity using 100 µl sample per reaction.[3] A third aliquot was washed 5 times with 1 ml MBH buffer each by using a 0.2 µm regenerated cellulose syringe filter (low protein binding). The filtrate was concentrated by ultrafiltration (50 kDa molecular weight cutoff) to ~200 µl and subsequently analyzed for catalytic H_2 oxidation activity using 100 µl sample per reaction.[3] Despite the content of ~63 µg of MBH per starting aliquot, activity was observed neither for the stirred, nor for the filtered aliquot. In control experiments an amount of 1.6 µg MBH leads to a slope of 1 min^{-1} and even the activity of ~10 ng MBH would be detectable. This indicated that there was no detectable leaching from the COF.

As an ultimate test, fractions of the last aliquot of MBH@TpPa COF composite, equal to 12 and 24 µg MBH, were pelleted (21,100 × g, 5 min, 4 °C) and the pellets were resuspended with SDS-PAGE sample buffer and heated to 95 °C for 2 minutes to unfold and extract the protein from the TpPa COF. As control, pure MBH protein (1, 5 and 15 µg) was mixed with sample buffer and heated as before. As COF controls, macroporous COF, loaded with MBH (1 µg and 5 µg) after COF synthesis, as described in a previous publication, was pelleted as described for the *in situ* synthesized MBH@TpPa COF. All samples were separated on a 4-20 % acrylamide SDS-PAGE and stained with colloidal Coomassie stain.[10] This staining method can detect amounts as low as 1 ng protein per band.

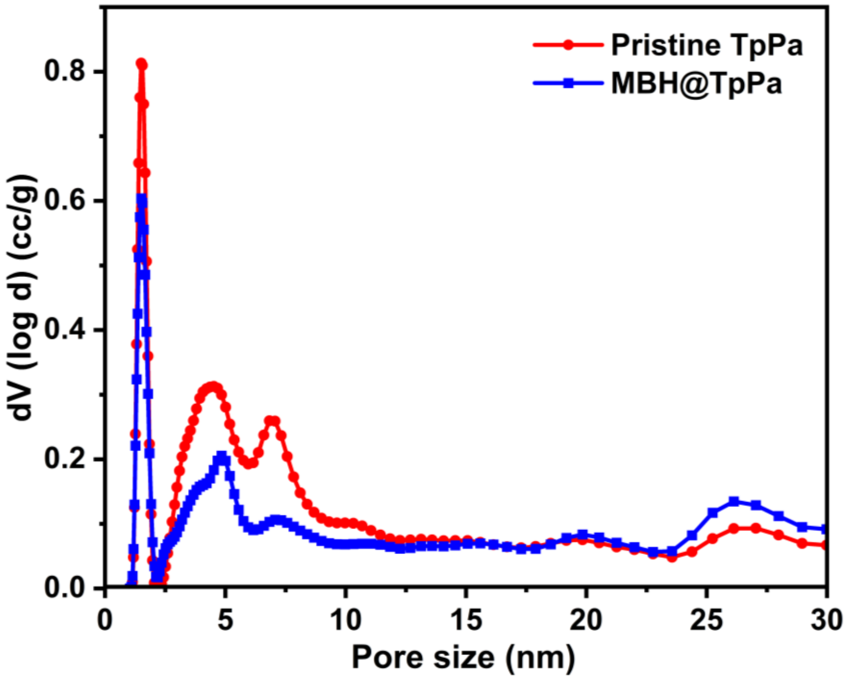

Fig. S1. *Pore size distribution analysis. Quenched solid density functional theory (QSDFT) pore size distributions calculated from the adsorption branches of the N_2 isotherms for pristine TpPa and MBH@TpPa COFs.*

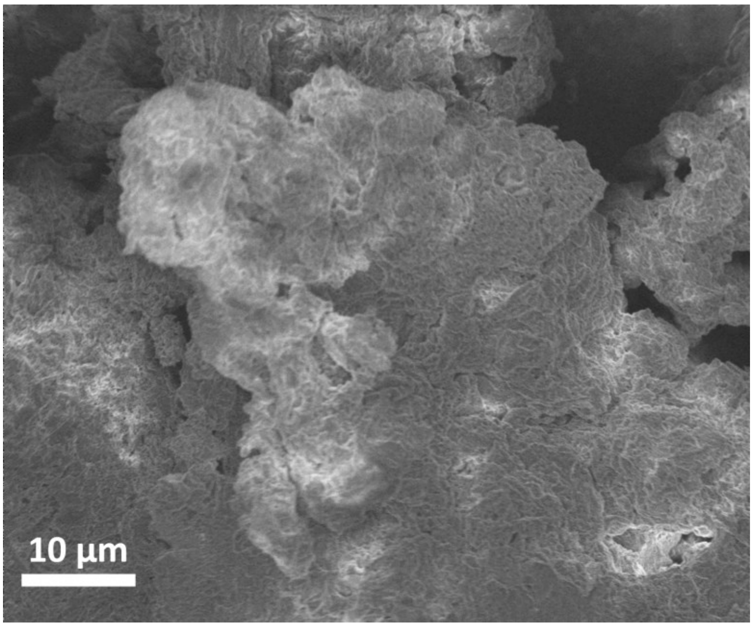

Fig. S2. *SEM images of MBH@TpPa. The COF forms solid, aggregated nanoparticles with no hollow interior.*

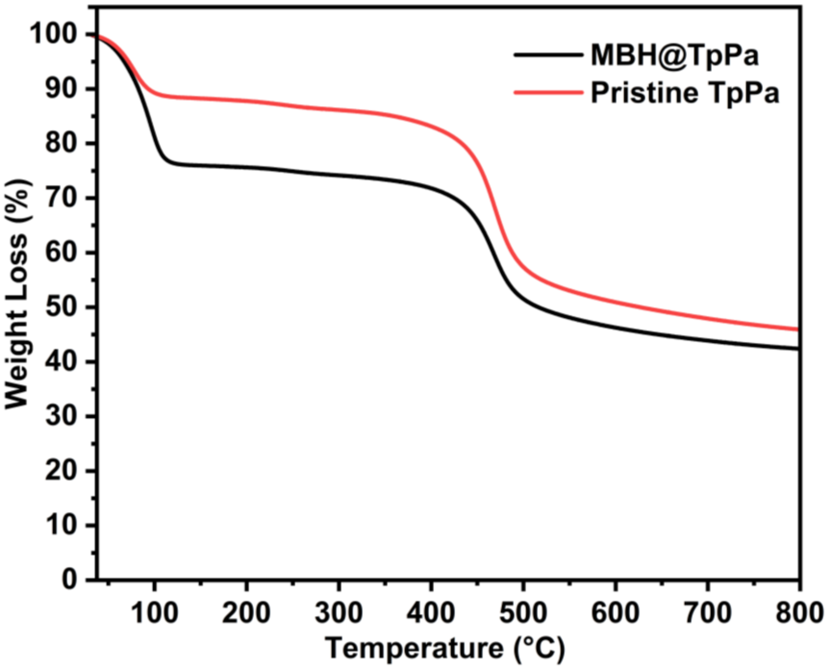

Fig. S3*. Thermogravimetric analysis (TGA). TGA profiles of as-synthesized MBH@TpPa and pristine TpPa COFs recorded under a* N_2 *atmosphere. The initial mass loss below 100 °C is attributed to the evaporation of residual water.*

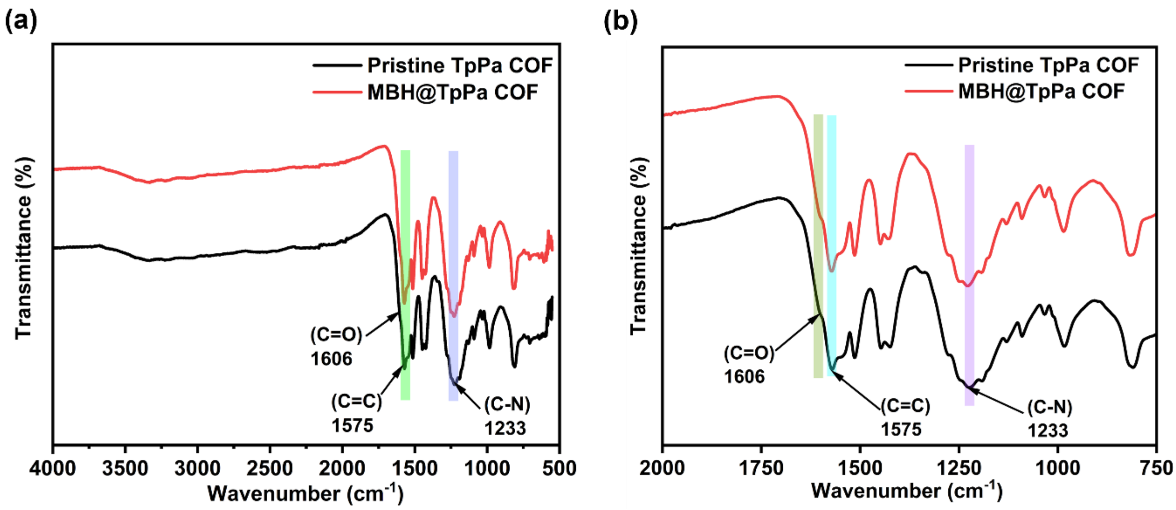

Fig. S4*. Fourier-transform infrared (FTIR) spectroscopy. a, Full-range FTIR spectra of MBH@TpPa and pristine TpPa COFs. b, Magnified view of the key spectral regions from panel a.*

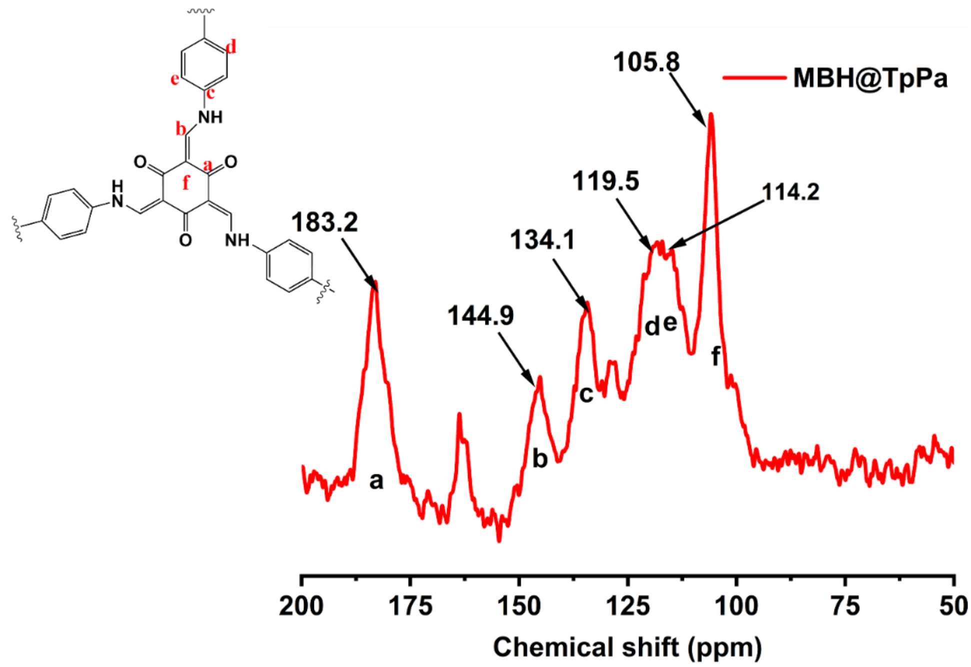

Fig. S5 *Solid-state ^{13}C nuclear magnetic resonance (NMR) spectroscopy. ^{13}C cross-polarization magic-angle spinning (CP/MAS) NMR spectrum of the MBH@TpPa composite. Minor unresolved signals, particularly in the 160–170 ppm region, are attributed to residual or defect-associated carbonyl/imine species, while broad features in the aromatic region arise from overlapping carbon environments within the TpPa framework.*

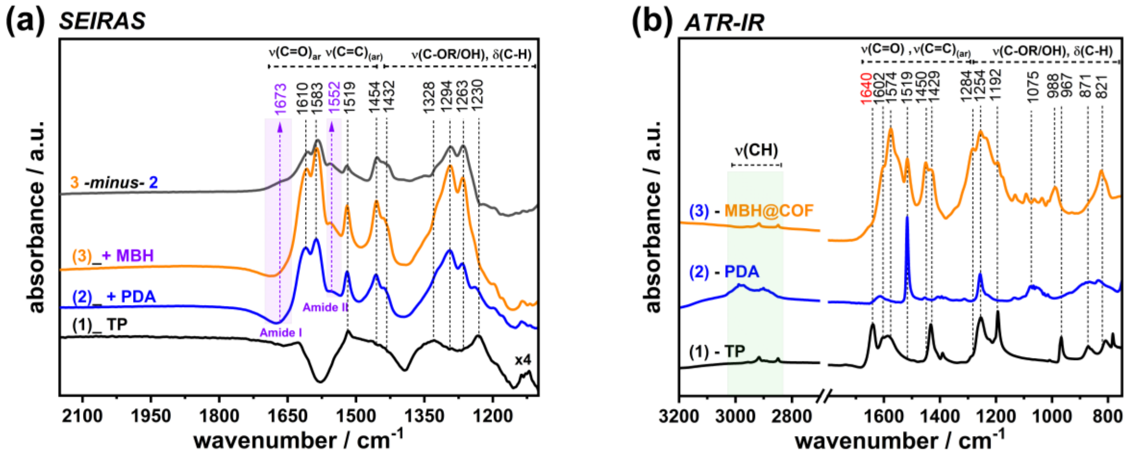

Fig. S6. *In situ spectroscopic monitoring of biohybrid formation. a, Stepwise formation of MBH@TpPa monitored by surface-enhanced infrared absorption spectroscopy (SEIRAS). Spectrum 1 (black) shows the signal from Tp near the gold surface (×4). Spectrum 2 (blue) was recorded after adding p-phenylenediamine (PDA). Spectrum 3 (orange) was taken after the subsequent addition of MBH, acetic acid, imidazole, ethanol, and final buffer exchange. Bands characteristic of the MBH protein backbone are highlighted in violet.*[11] *b, Attenuated total reflectance IR (IR-ATR) spectra of the Tp monomer (1, black), PDA monomer (2, blue), and the final MBH@TpPa COF (3, orange). Characteristic spectral regions are indicated.*

Table S3. *Quantification of MBH loading via metal analysis. Determination of MBH enzyme loading within the TpPa COF matrix based on inductively coupled plasma mass spectrometry (ICP-MS) measurement of the [NiFe] cofactor metals.*

ng metal/mg COF	*Pristine TpPa*	*MBH@TpPa*	*MBH@TpPa corrected for pristine TpPa background*
Ni	*0.16 ± 0.07*	*1.40 ± 0.05*	*1.24 ± 0.08*
Fe	*3.17 ± 0.30*	*15.34 ± 0.16*	*12.17 ± 0.34*
Molar ratio Fe/Ni	*21.4*	*11.5*	*10.3*

Table S4. *Corrected physicochemical parameters for MBH@TpPa. Key textural and electronic properties of the MBH@TpPa biohybrid, with values normalized to account for the contribution from the encapsulated enzyme mass.*

	wt% (in terms of the metal)	nmol MBH/mg COF (according to metal)
Ni	0.00012	0.021 ± 0.0014
Fe	0.00122	0.018 ± 0.0005

EDS (Energy Dispersive Spectroscopy) elemental mapping was not performed due to the extremely low Ni and Fe content (<0.002 wt%), which is several orders of magnitude below the typical detection limit of the technique (~0.5–1 wt%). Therefore, ICP-MS was used for quantitative analysis instead.

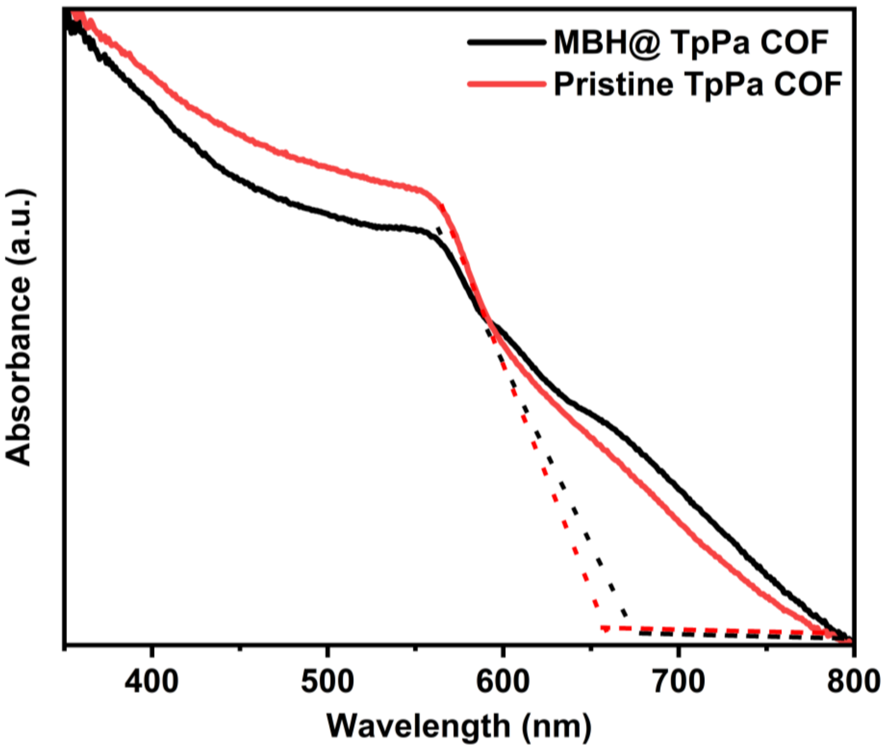

Fig. S7*. Solid-state UV-Vis spectroscopy. Diffuse reflectance UV-Vis spectra of MBH@TpPa and pristine TpPa COFs.*

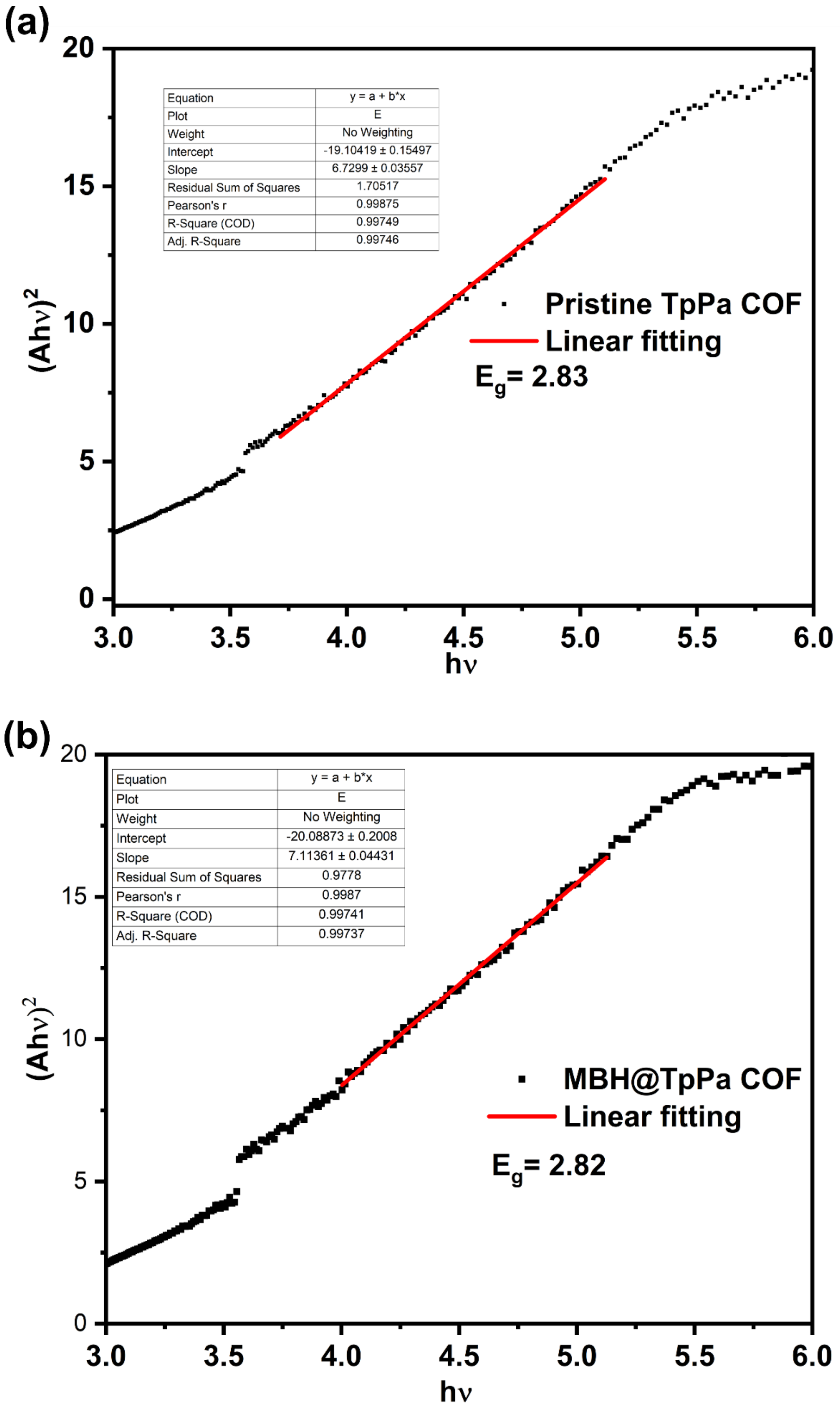

Fig. S8*. Optical band gap determination. Tauc plots derived from UV-Vis data for (a) pristine TpPa COF and (b) MBH@TpPa COF, indicating the optical band gap energy.*

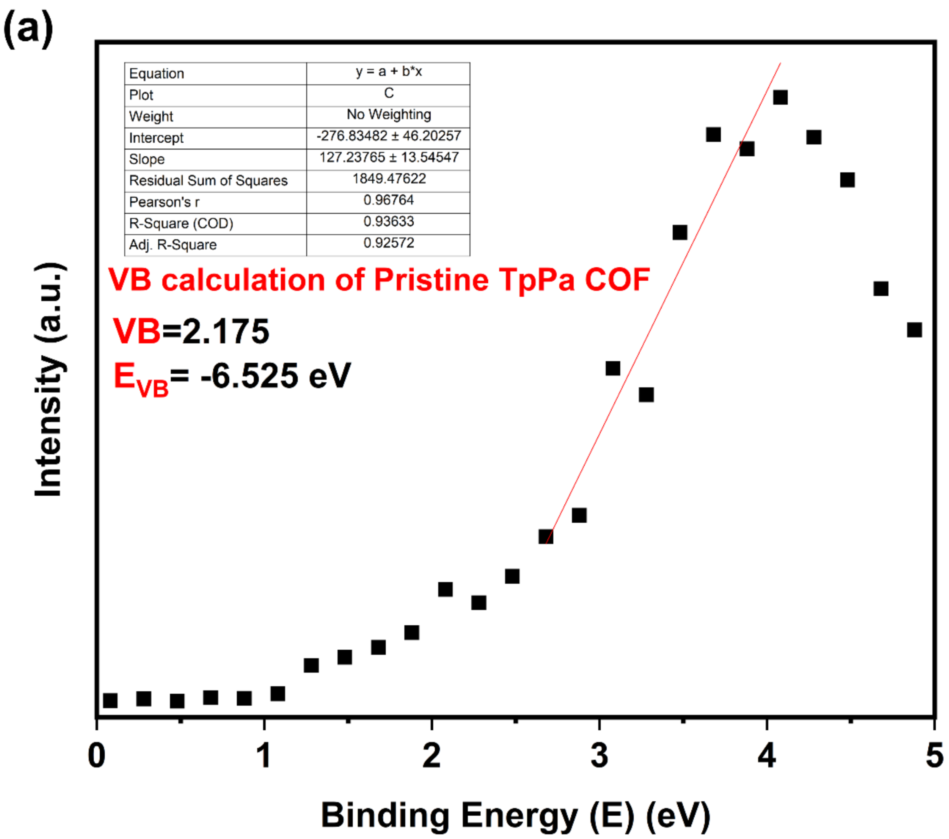

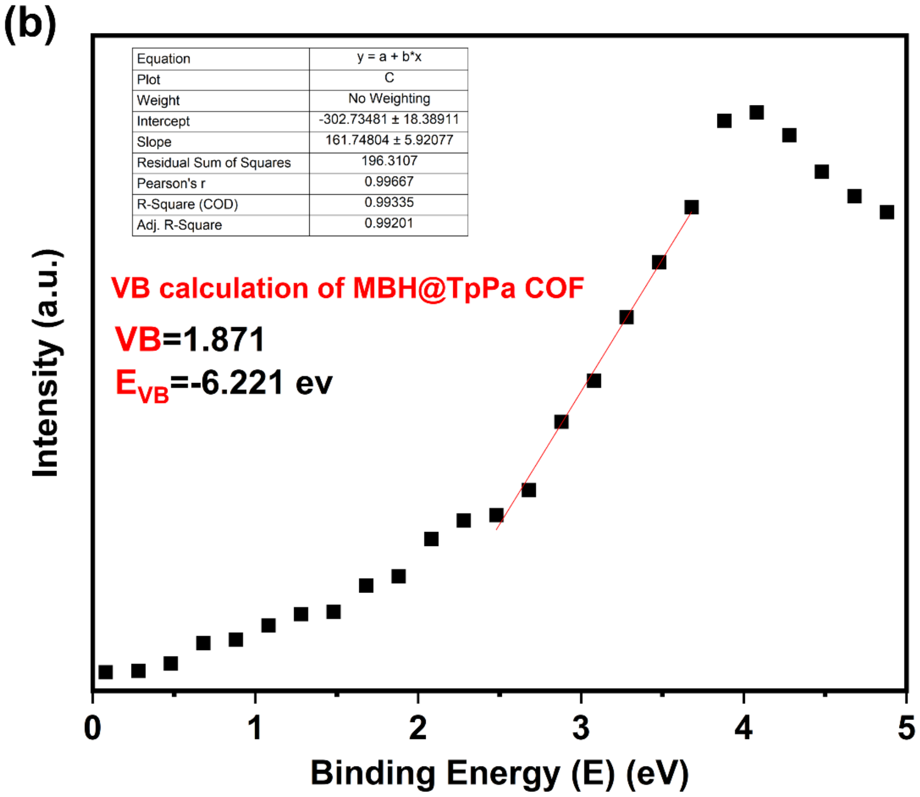

Fig. S9. *Valence band analysis. Valence band spectra from X-ray photoelectron spectroscopy (XPS) for (a) pristine TpPa COF and (b) MBH@TpPa COF.*

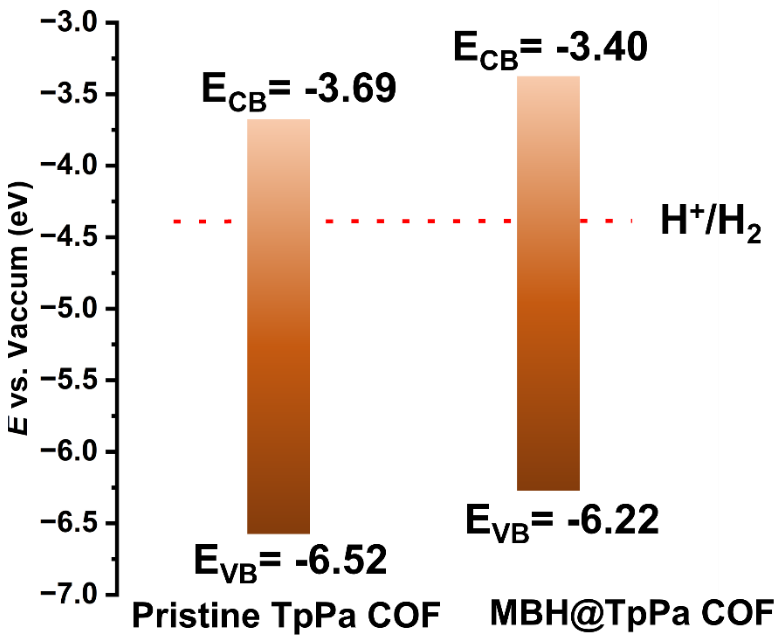

Fig. S10. *Electronic band structure diagram. Experimental band gap energies and relative band edge positions for MBH@TpPa and pristine TpPa COFs, constructed from UV-Vis and XPS data.*

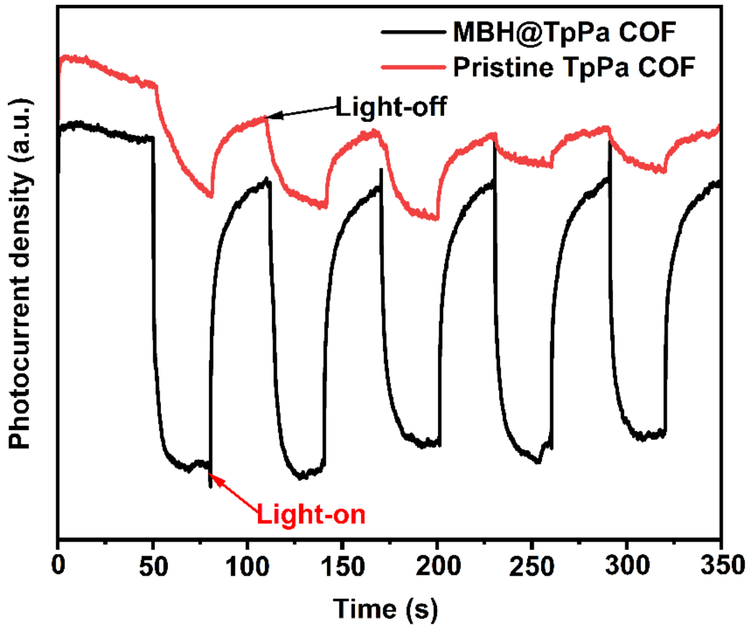

Fig. S11. *Transient photocurrent response. Current density versus time for (a) pristine TpPa COF and (b) MBH@TpPa COF under intermittent illumination.*

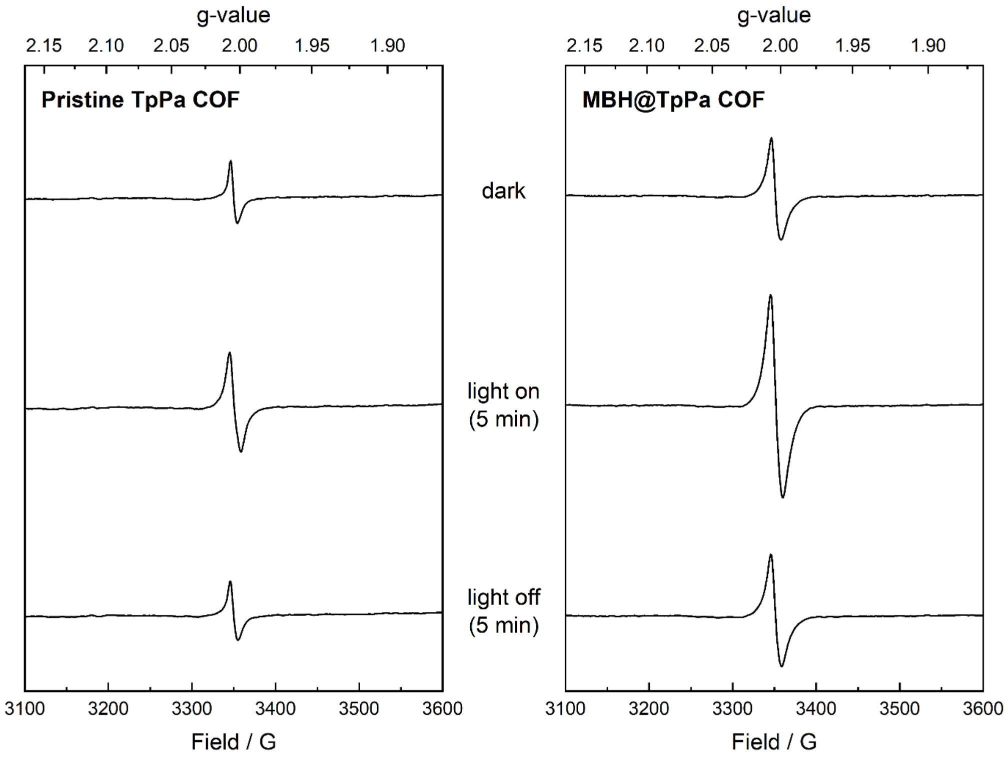

Fig. S12. *Light-dependent electron paramagnetic resonance (EPR) signals. EPR spectra of pristine TpPa COF (left) and MBH@TpPa COF (right) recorded at room temperature under three conditions: in the dark (top), after 5 minutes of illumination (middle), and 5 minutes after the light was switched off (bottom). Room temperature measurements enable the exclusive detection of the organic radical signals stemming from the COF material.*

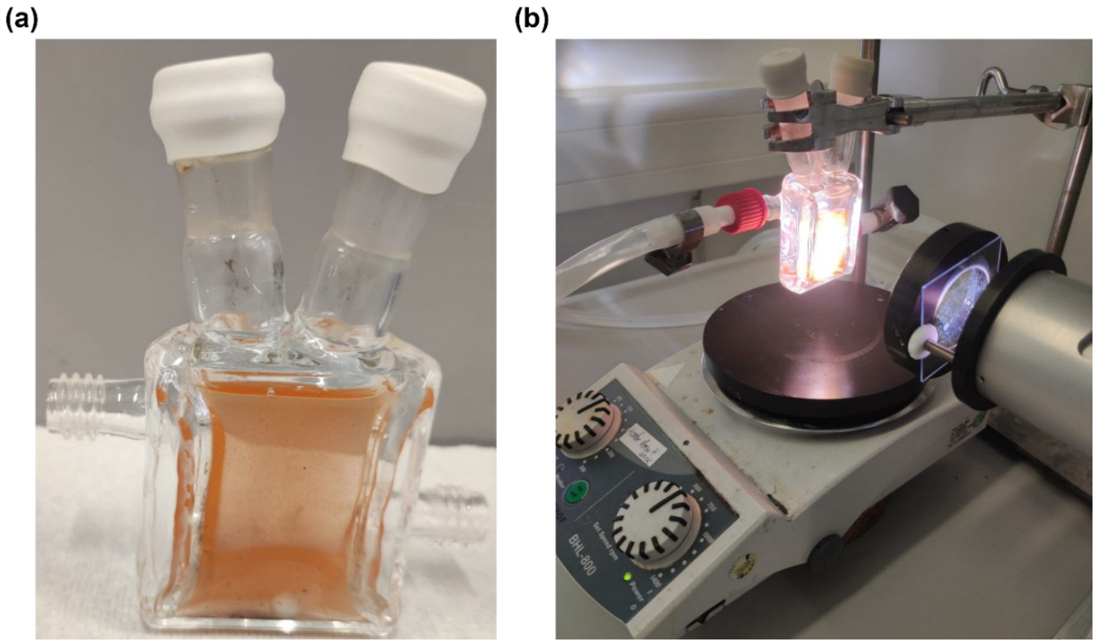

Fig. S13. *Photographic documentation of the photocatalytic setup. a, Photograph of the reactor containing the MBH@TpPa COF suspension during an experiment. b, Overview*

photograph of the full photocatalytic hydrogen production setup with the xenon lamp light source.

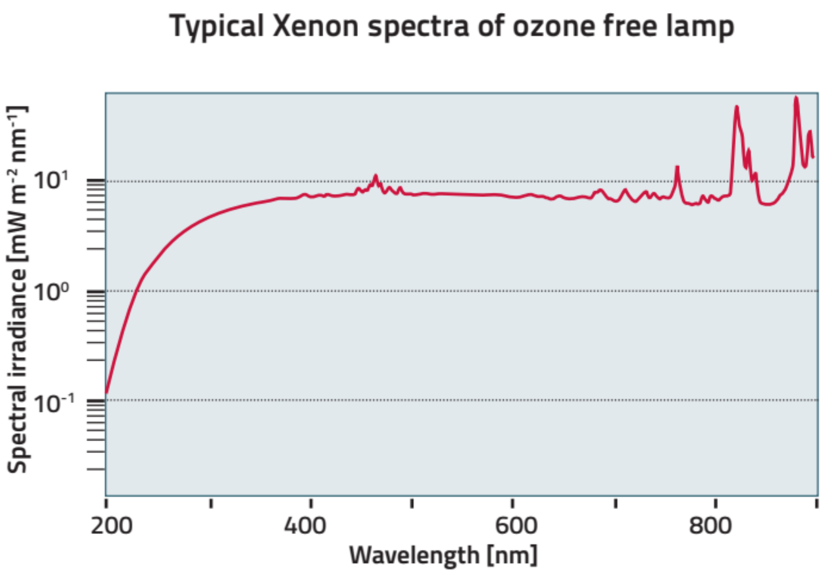

***Fig. S14**. Emission spectrum of the light source. Normalized output spectrum of the xenon lamp used for all photocatalytic experiments.*[12,13]

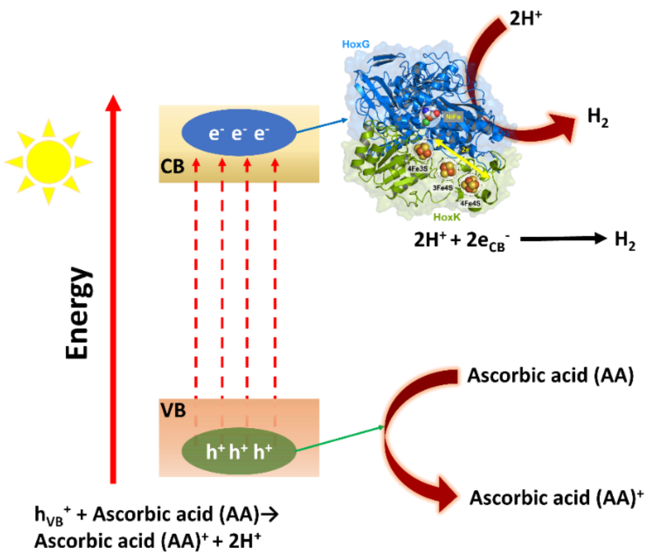

***Fig. S15**. Proposed mechanism for the photocatalytic hydrogen evolution reaction. The scheme illustrates the process using a sacrificial electron donor (ascorbic acid). VB (valence band), CB (conduction band)*

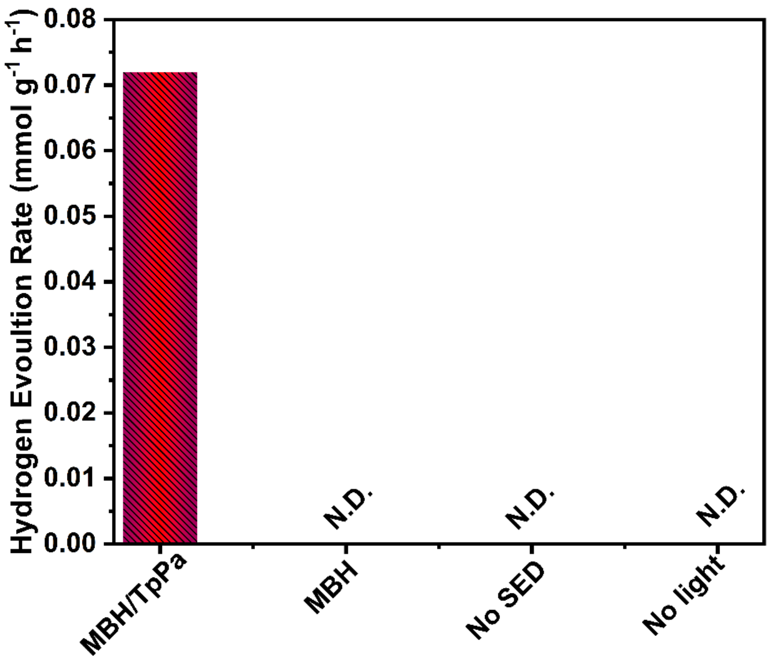

Fig. S16. *Control experiments for photocatalytic hydrogen evolution. Hydrogen evolution rates for a physical mixture of MBH and TpPa COF (MBH/TpPa), free MBH in solution, and the MBH@TpPa COF tested in the absence of a sacrificial electron donor (SED) or in the dark. "N.D." denotes not detectable activity.*

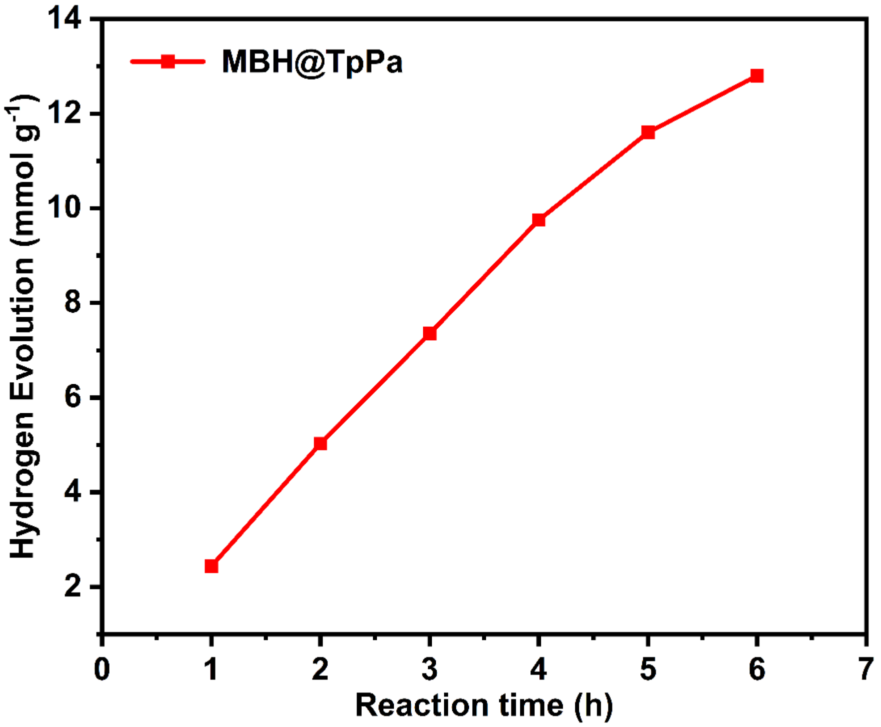

Fig. S17. *Long-term photocatalytic hydrogen evolution. Time-dependent hydrogen evolution activity of the MBH@TpPa biohybrid under continuous photocatalytic conditions.*

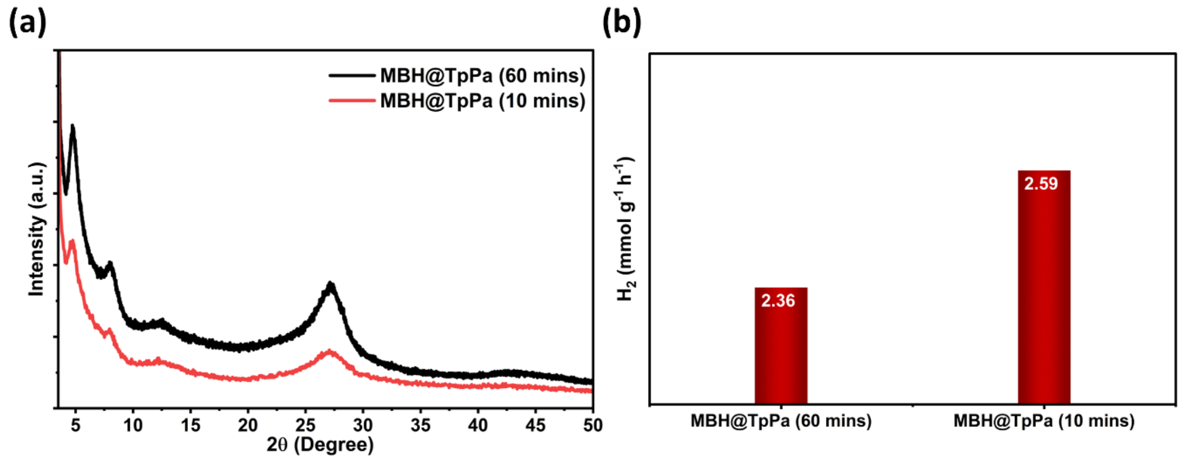

Fig. S18. *Effect of extending COF synthesis time on MBH@TpPa. (a) Powder XRD patterns (10 min, red; 1 h, black). The 1 h sample shows more pronounced peaks, indicating a modest increase in crystallinity. (b) Photocatalytic HER activity under identical conditions. The 1 h sample shows slightly lower activity (2.36 mmol g^{-1} h^{-1}) compared to the 10 mins. sample (2.59 mmol g^{-1} h^{-1}), confirming that longer synthesis does not enhance performance.*

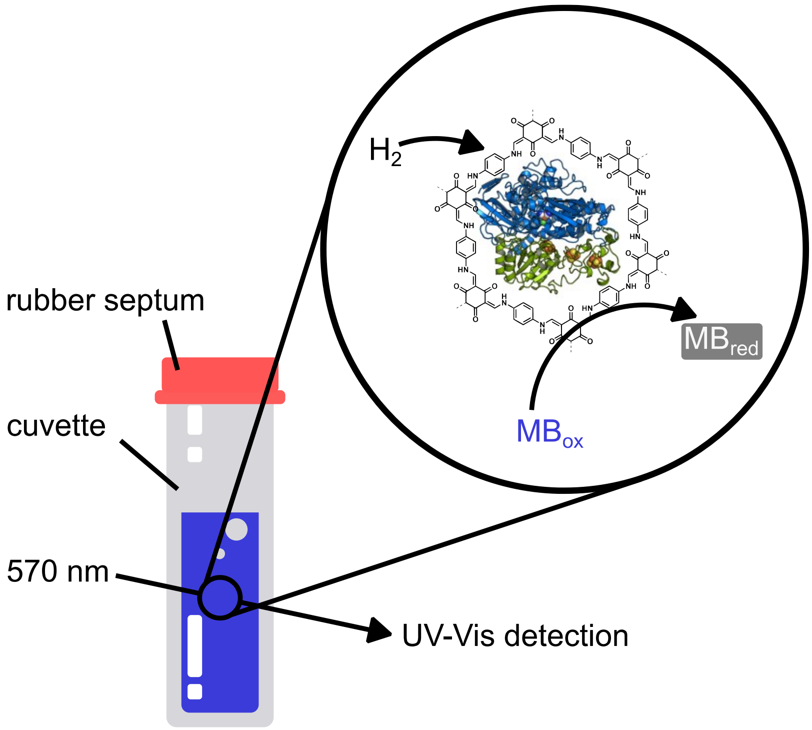

Fig. S19. *Schematic of the hydrogen oxidation activity assay. The assay was performed in a sealed cuvette. The COF sample in buffer was saturated with H_2 gas, after which the reaction was initiated by injecting the electron acceptor methylene blue (MB_{ox}, blue). MBH-catalyzed H_2 oxidation reduces MB_{ox} to its colorless form (MB_{red}), which is monitored by the decrease in absorbance at 570 nm.*

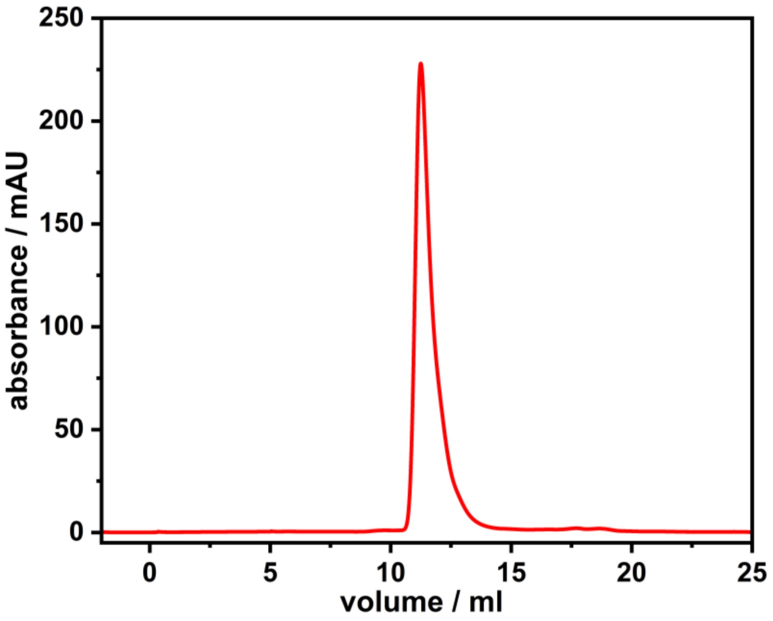

Fig. S20. *Protein purification analysis. Size-exclusion chromatography trace confirming the buffer exchange of MBH from phosphate to imidazole buffer. MBH elutes as a single, narrow peak, indicating sample purity and homogeneity.*

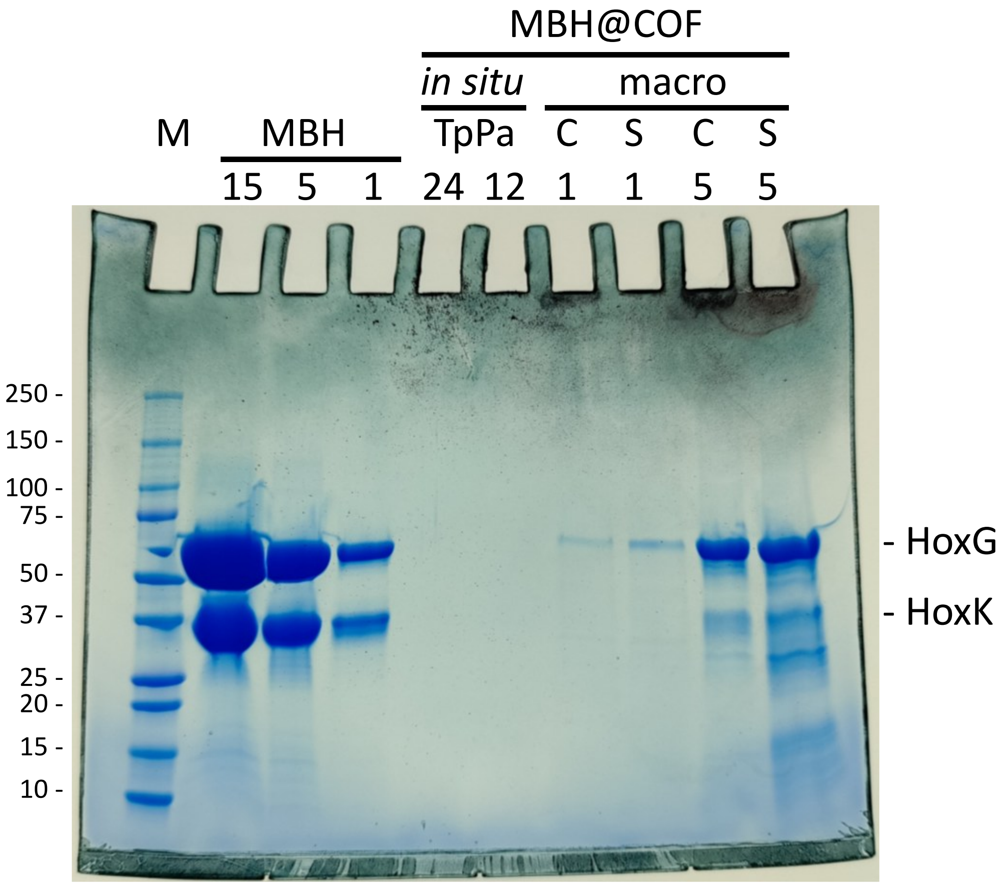

Fig. S21. *Leaching test of MBH@TpPa COF. Amounts of in situ-synthesized MBH@TpPa COF corresponding to 12 and 24 µg of MBH were resuspended in SDS-PAGE sample buffer and heated to 95 °C for 2 minutes to enable unfolding and detachment of the MBH subunits, and the samples were separated on a 4-20 % acrylamide SDS-PAGE. The gel was stained using an established staining protocol that detects protein amounts as low as 1 ng per protein band.*[10]

As reference, 1, 5 and 15 µg of MBH were loaded on the same gel to illustrate the positions and intensities of the subunits on the gel. As control, macroCOF (macro) loaded with MBH after COF synthesis,[14] *was analyzed in parallel. The amounts of protein loaded are indicated on top of the gel. Molecular masses are indicated in kDa on the left-hand side. S and C indicate SO_3- and COOH-modified macroCOF, respectively. In case of the macroCOF samples, degradation of the small MBH subunit HoxK is visible which is presumably caused by the long-term storage at 4 °C over several months. Furthermore, the lower protein content indicates that a fraction of protein was lost over time.*

Supplementary Table S5. Comparison of the hydrogen evolution rate (HER) achieved by the MBH@TpPa biohybrid with other representative COF-based and hybrid photocatalysts under visible light irradiation. Key reaction parameters; including light source, sacrificial electron donor, catalyst loading, apparent quantum efficiency (AQE), and activity, are provided for each entry to enable direct comparison.

COFs	Cocatalyst	Sacrificial agent	Illumination	Activity (mmol g^{-1} h^{-1})	AQE (%)	Ref
**MBH@TpPa COF**	**MBH**	**Ascorbic acid / Sodium ascorbate/water**	**> 420 nm**	**2.59**	**0.27 %**	**This work**
N3–COF	8wt% Pt	TEOA	> 420 nm	1.703	**0.44 %**	*Nat. Commun.* **2015**, 6, 8508
Py-HTP-BT-COF	5wt % Pt	Ascorbic acid	> 420 nm	1.078	-----	*Angew. Chem. Int. Ed. Engl.* **2020**, *59*, 16902-16909
Py-FTP-BT-COF	5wt % Pt	Ascorbic acid	> 420 nm	2.875	------	*Angew. Chem. Int. Ed. Engl.* **2020**, *59*, 16902-16909
ZnPor-DETH-COF	8wt% Pt	TEOA	> 420 nm	0.413	0.063 %	*Nat. Commun.* **2021**, *12*, 1354

TpPa-2-COF	α-Fe_2O_3	Sodium Ascorbate	> 420 nm	3.770	0.137 %	*J. Mater. Chem. A.* **2020**, *8*, 4334-4340
TpPa-2-COF	Cu-NH_2-MIL-125	Sodium Ascorbate	> 420 nm	9.210	8.6%	*Appl. Catal. B-Environ.* **2022**, *317-121710*
BtCOF150	1wt% Pt	TEOA	> 420 nm	0.750	0.2%	*J. Am. Chem. Soc.* **2020**, *142*, 9752–9762
FS-COF	8wt % Pt	Ascorbic acid	> 420 nm	10.100	-----	*Nat. Chem.* **2018**, *10*, 1180-1189
g-$C_{18}N_3$–COF	3wt % Pt	Ascorbic acid	> 420 nm	0.292	1.06 %	*J. Am. Chem. Soc.* **2019**, *141*, 14272-14279
TtaTfa_AC	8wt % Pt	Ascorbic acid	> 420 nm	20.700	1.43 %	*Angew. Chem. Int. Ed. Engl.* **2021**, *60*, 19797-19803
Pt_1@TpPa-1	0.72 wt% Pt	Sodium Ascorbate	> 420 nm	0.719	0.38 %	*ACS Catal.* ***2021**, 11, 21, 13266–13279*
DCNA-1-AC	8wt % Pt	Ascorbic acid	> 420 nm	27.900	1.49 %	*Nat. Commun.* **2022**, *13*, 6317

Tp-Py COF (nonstoichiometric)	None	Ascorbic acid	>420 nm	22.45 (pure water); 15.48 (seawater)	3.3%	*Nat. Commun.* ***2025**, 16, 3024*
Py-PaCN COF	3wt % Pt	Ascorbic acid	>420 nm	12.1	7.15 %	*J. Mater. Chem. A, 2025,13, 14304-14313*
PyCN-Pa COF	3wt % Pt	Ascorbic acid	>420 nm	4.3	2.54 %	*J. Mater. Chem. A, 2025,13, 14304-14313*
TpPaCl	None	Ascorbic acid	>420 nm	16.3	2.12 %	*Chem. Sci. 2025, 16, 12906*
TpDTz 2D-COF	Cobalt (1% Co w/w)	Sodium ascorbate : Ascorbic acid 1:1	6 400 (AM 1.5G) 10 400 (LED 525 nm, 100 mW·cm-2)	10.4	----	*J. Am. Chem. Soc.* ***2026**, 148, 1, 1316–1328*

Reference

[1] J. H. Chong, M. Sauer, B. O. Patrick, M. J. Maclachlan, "Highly Stable Keto-Enamine Salicylideneanilines" *Org. Lett.* **2003**, *5*, 3823–3826.

[2] L. Guo, Y. Zhang, Z. Yu, R. Krishna, F. Luo, "Minute and Large-Scale Synthesis of Covalent-Organic Frameworks in Water at Room Temperature by a Two-Step Dissolution−Precipitation Method" *Chem. Mater* **2023**, *35*, 5656.

[3] O. Lenz, L. Lauterbach, S. Frielingsdorf, "O2-tolerant [NiFe]-hydrogenases of Ralstonia eutropha H16: Physiology, molecular biology, purification, and biochemical analysis" *Methods Enzymol.* **2018**, *613*, 117–151.

[4] C. Lorent, Combining Electron Paramagnetic Resonance and Vibrational Spectroscopy to Explore Catalysis of Hydrogen-Converting Enzymes, **2022**.

[5] J. Fritsch, P. Scheerer, S. Frielingsdorf, S. Kroschinsky, B. Friedrich, O. Lenz, C. M. T. Spahn, "The crystal structure of an oxygen-tolerant hydrogenase uncovers a novel iron-sulphur centre" *Nature* **2011**, *479*, 249–253.

[6] H. Miyake, S. Ye, M. Osawa, "Electroless deposition of gold thin films on silicon for surface-enhanced infrared spectroelectrochemistry" *Electrochem. commun.* **2002**, *4*, 973–977.

[7] M. Osawa, "Dynamic Processes in Electrochemical Reactions Studied by Surface-Enhanced Infrared Absorption Spectroscopy (SEIRAS)" *Bull. Chem. Soc. Jpn.* **1997**, *70*, 2861–2880.

[8] A. Barth, "Infrared spectroscopy of proteins" *Biochim. Biophys. Acta - Bioenerg.* **2007**, *1767*, 1073–1101.

[9] A. Limbeck, M. Bonta, W. Nischkauer, "Improvements in the direct analysis of advanced materials using ICP-based measurement techniques" *J. Anal. At. Spectrom.* **2017**, *32*, 212–232.

[10] G. Candiano, M. Bruschi, L. Musante, L. Santucci, G. M. Ghiggeri, B. Carnemolla, P. Orecchia, L. Zardi, P. G. Righetti, "Blue silver: A very sensitive colloidal Coomassie G-250 staining for proteome analysis" *Electrophoresis* **2004**, *25*, 1327–1333.

[11] A. Yarman, A. F. T. Waffo, S. Katz, C. C. M. Bernitzky, N. Kovács, P. Borrero, S. Frielingsdorf, E. Supala, J. Dragelj, S. Kurbanoglu, B. Neumann, O. Lenz, R. E. Gyurcsányi, M.-A. Mroginski, U. Wollenberger, F. W. Scheller, G. Caserta, I. Zebger, "A Strep-Tag Imprinted Polymer Platform for Heterogenous Bio(electro)catalysis" *Angew. Chemie Int. Ed.* **2024**, *63*, e202408979.

[12] I. E. Khalil, P. Das, H. Küçükkeçeci, V. Dippold, J. Rabeah, W. Tahir, J. Roeser, J. Schmidt, A. Thomas, "Hierarchical Porous Covalent Organic Frameworks: The Influence of Additional Macropores on Photocatalytic Hydrogen Evolution and Hydrogen Peroxide Production" *Chem. Mater.* **2024**, *36*, 8330−8337.

[13] P. Das, G. Chakraborty, J. Yang, J. Roeser, H. Küçükkeçeci, A. D. Nguyen, M. Schwarze, J. Gabriel, C. Penschke, S. Du, V. Weigelt, I. E. Khalil, J. Schmidt, P. Saalfrank, M. Oschatz, J. Rabeah, R. Schomäcker, F. Emmerling, A. Thomas, "The Effect of Pore Functionality in Multicomponent Covalent Organic Frameworks on Stable Long-Term Photocatalytic H2

Production” *Adv. Energy Mater.* **2025**, *16*, 2501193.

[14] I. E. Khalil, A. F. T. Waffo, P. Das, S. Katz, S. Kunow, C. Lorent, Y. Ziouani, W. Tahir, A. A. Owusu, O. Lenz, I. Zebger, S. Frielingsdorf, A. Thomas, “Enhancing and Stabilizing Hydrogen Catalysis Through [NiFe]-Hydrogenase Immobilization Within Macroporous Covalent Organic Frameworks” *Adv. Funct. Mater.* **2025**, *36*, e20237.
